# Synthesis of Novel Benzazole Derivatives and Evaluation of Their Antidepressant-Like Activities with Possible Underlying Mechanisms

**DOI:** 10.3390/molecules23112881

**Published:** 2018-11-05

**Authors:** Gamze Tokgöz, Ümide Demir Özkay, Derya Osmaniye, Nazlı Turan Yücel, Özgür Devrim Can, Zafer Asım Kaplancıklı

**Affiliations:** 1 Department of Pharmacology, Faculty of Pharmacy, Anadolu University, Eskişehir 26470, Turkey; eczgamzetokgoz@gmail.com (G.T.); nazlituran@anadolu.edu.tr (N.T.Y.); ozgurdt@anadolu.edu.tr (Ö.D.C.); 2 Department of Pharmaceutical Chemistry, Faculty of Pharmacy, Anadolu University, Eskişehir 26470, Turkey; dosmaniye@anadolu.edu.tr (D.O.); zakaplan@anadolu.edu.tr (Z.A.K.); 3 Doping and Narcotic Compounds Analysis Laboratory, Faculty of Pharmacy, Anadolu University, Eskişehir 26470, Turkey

**Keywords:** activity cage test, antidepressant, benzazole, modified forced swimming test, tail suspension test

## Abstract

Novel benzazole derivative compounds **4a**–**4h** were obtained by the reaction of corresponding 2-(benzazol-2-ylthio)acetohydrazide and appropriate 4-substituted benzaldehydes. The chemical structures of the synthesized compounds were elucidated by FT-IR, ^1^H-NMR, ^13^C-NMR and LCMS spectroscopic methods. Antidepressant-like effects of the compounds were evaluated by tail suspension test (TST) and modified forced swimming tests (MFST). Moreover, locomotor activities of the animals were assessed by an activity cage apparatus. In the series, compounds **4a**, **4b**, **4e** and **4f** (at 50 mg/kg) significantly decreased the immobility time of mice in both of the TST and MFST. The same compounds prolonged the swimming time of animals in MFST without any change in the climbing duration. These data indicated that compounds **4a**, **4b**, **4e** and **4f** possess significant antidepressant-like activities. Moreover, pre-treatments with *p*-chloro-phenylalanine methyl ester (an inhibitor of serotonin synthesis), NAN-190 (a 5-HT_1A_ antagonist), ketanserin (a 5-HT_2A/2C_ antagonist), and ondansetron (a 5-HT_3_ antagonist) reversed the exhibited pharmacological effects. Results of the mechanistic studies suggested the involvement of serotonergic system and contributions of 5-HT_1A_, 5-HT_2A/2C_ and 5-HT_3_ receptors to the antidepressant-like effects of compounds **4a**, **4b**, **4e** and **4f**. Furthermore, unchanged locomotor activity of mice following the administrations of these four derivatives confirmed that the presented antidepressant-like effects are specific.

## 1. Introduction

Depression is a common and incapacitating mental health condition that is estimated to affect over 300 million people worldwide. The main symptoms of depression are sad mood, loss of interest or pleasure, low self-worth, feel of guilty, lack of concentration, anhedonia, suicidal thoughts, disrupted sleep, appetite or energy [1,2]. Clinically used antidepressant drugs such as selective serotonin reuptake inhibitors, tricyclic antidepressants, and monoamine oxidase inhibitors alleviate the symptoms of major depression. However low response rates, delayed onset of therapeutic action or various side effects associated with the use of current antidepressants are still major challenges in the management of this disease limiting the success of treatment and causing compliance problems in patients [3,4]. Therefore, studies for discovering and developing new antidepressant drugs are of great importance.

The benzazole type compounds such as benzimidazoles and benzothiazoles are frequently preferred rings in drug development studies because of their widespread pharmacological activity potential. For example, various benzimidazole-based compounds have been reported for their antibacterial [5], anticancer [6], anti-HIV [7], anti-ulcer [8], anti-asthmatic [9], antidiabetic [10,11], anti-inflammatory [11], antihypertensive [12], cholinesterase inhibitory, and anti-oxidant [13] effects. Numerous benzothiazole derivatives have also been shown to induce antibacterial, antifungal, anticancer [14], anti-oxidant [15], antiviral [16], antituberculosis [17], antidiabetic [18], and anti-inflammatory [19] activities, so far.

Various compounds bearing benzimidazole or benzothiazole ring systems in their chemical structures have been demonstrated to possess potential for passing through the blood brain barrier and triggering some central nervous system (CNS)-related pharmacological activities. Actually, benzimidazole derivative compounds have been shown to induce antidepressant-like [20,21,22], antinociceptive [23,24] and anticonvulsant [10,11,25] activities. The same activities [19,26,27,28,29,30,31,32] as well as anti-Alzheimer [33,34] and neuroprotective [35] effects have also been reported for compounds carrying benzothiazole groups. Therefore, based on the CNS-related activity potential of benzimidazole and benzothiazole ring systems, in the present study, we synthesized some novel compounds carrying both of these ring systems on their chemical scaffolds and investigated their potential antidepressant-like activities using some well-known in vivo methods.

## 2. Results and Discussion

### 2.1. Chemistry

**Scheme 1:**
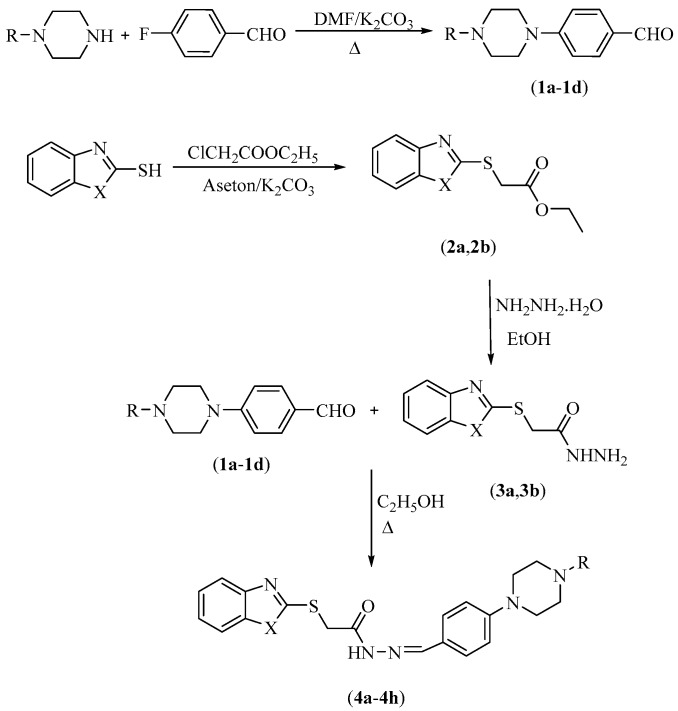
Synthesis of the target compounds **4a**–**4h**.

The compounds were obtained through a four-step synthesis as presented in Scheme 1. Initially, 4-substituted benzaldehyde derivatives **1a**–**1h** were prepared by refluxing 4-fluorobenzaldehyde and appropriate secondary amines in DMF. Secondly, ethyl 2-(benzazol-2-yl-thio)acetates (**2a**,**2b**) were gained as a result of reactions between an appropriate benzazol-2-thiol and ethyl 2-chloroacetate. Thirdly, the reaction of compounds **2a**,**2b** and excess of hydrazine hydrate afforded 2-((5-substitutedbenzazol-2-yl)thio)acetohydrazides (**3a**,**3b**), which were reacted with the appropriate 4-substituted benzaldeydes **1a**–**1h** to afford the target compounds **4a**–**4h**. Structure elucidations of the final compounds were performed by IR, ^1^H-NMR, ^13^C-NMR, and MS spectroscopic methods (see Supplementary Materials).

### 2.2. Pharmacology

In this study, antidepressant-like effects of the test compounds were investigated with tail suspension test (TST) and modified forced swimming tests (MFST), which are commonly used methods in antidepressant-like activity screening studies [36,37,38,39]. 

**Figure 1:**
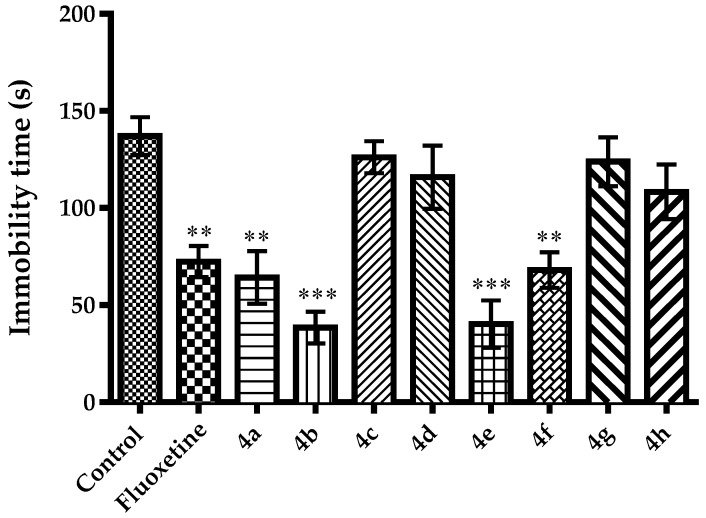
The effect of **4a**–**4h** (50 mg/kg) and fluoxetine (20 mg/kg) on immobility time of animals in the tail suspension test. ** *p* < 0.01, *** *p* < 0.001, compared with control group; One way analysis of variance followed by Tukey HSD multiple comparison test, n = 7.

In the TST, animals are hanged from their tails. Under this short-term and inescapable stressful condition, animals become immobile following some escape-oriented movements. In this test, the duration of “immobile posture” reflecting the behavioral despair is measured. It is known that various antidepressant drugs reduce the immobility time of animals and increase the escape-related movements in the TST [40,41,42,43]. The effects of the tested compounds and reference drug fluoxetine on the immobility time of mice in the TST are illustrated in Figure 1 [F(9,60) = 10.27, *p* < 0.001]. Tukey HSD multiple comparison tests following one-way ANOVA demonstrated that compounds **4a**, **4b**, **4e**, **4f** and reference drug fluoxetine significantly reduced the immobility time of animals pointing out the antidepressant-like effects of these compounds.

**Figure 2:**
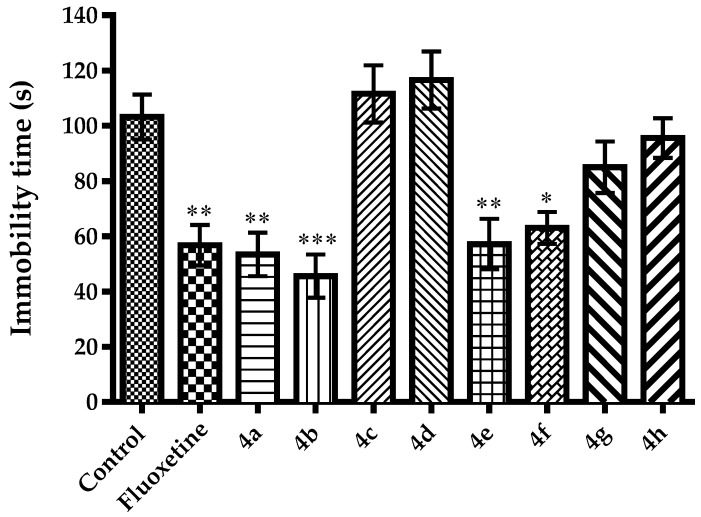
The effect of **4a**–**4h** (50 mg/kg) and fluoxetine (20 mg/kg) on immobility time of animals in the modified forced swimming test. * *p* < 0.05, ** *p* < 0.01, *** *p* < 0.001, compared with control group; One way analysis of variance followed by Tukey HSD multiple comparison test, n = 7.

MFST, a modified version of Porsolt’s forced swimming test, is based on the behavioral analysis of animals that are forced to swim in an inescapable narrow cylinder. Animals struggle to escape from this environment and after a while, they develop an immobile posture. This immobility behavior is considered as “behavioral despair”. Various antidepressant drugs have been reported to reduce immobility times of rodents in MFST [42,43,44]. In this study, effects of the test compounds and fluoxetine on immobility time of mice in the MFST was shown in Figure 2 [F(9,60) = 9.95, *p* < 0.001]. Results of the post-hoc analysis indicated that compounds **4a**, **4b**, **4e**, **4f** and fluoxetine caused significant decreases in the immobility time of mice in MFST. Findings of the MFST, revealing the antidepressant-like effects of compounds **4a**, **4b**, **4e** and **4f**, supported the data obtained from the TST.

**Figure 3:**
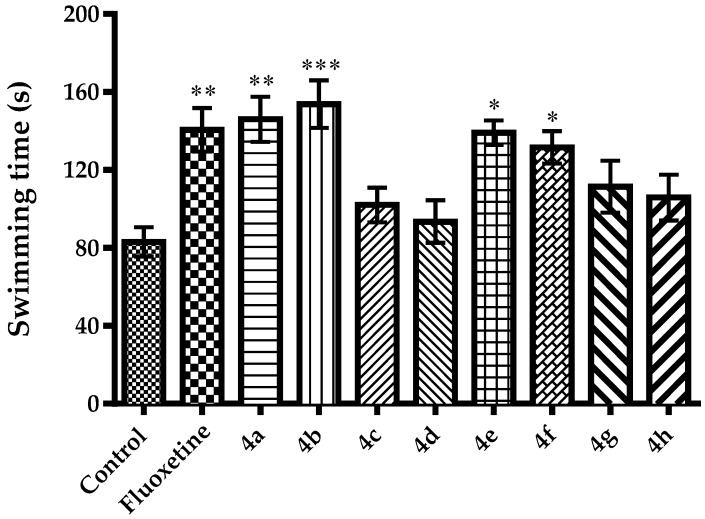
The effect of **4a**–**4h** (50 mg/kg) and fluoxetine (20 mg/kg) on swimming time of animals in the modified forced swimming test. * *p* < 0.05, ** *p* < 0.01, *** *p* < 0.001, compared with control group; One way analysis of variance followed by Tukey HSD multiple comparison test, n = 7.

**Figure 4:**
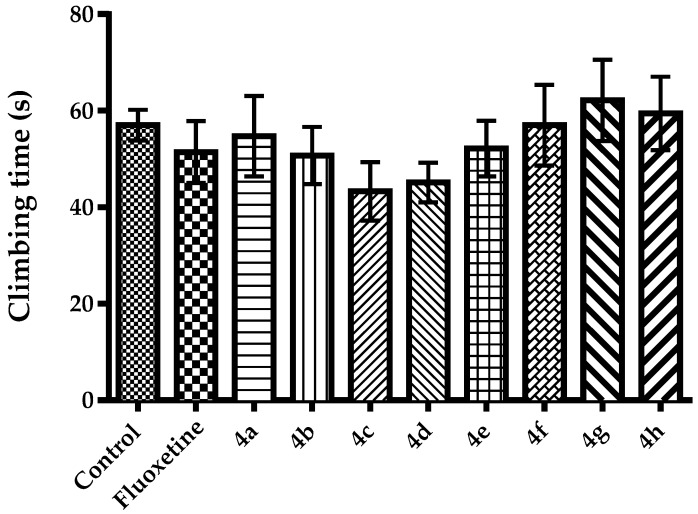
The effect of **4a**–**4h** (50 mg/kg) and fluoxetine (20 mg/kg) on climbing time of animals in the modified forced swimming test. One way analysis of variance followed by Tukey HSD multiple comparison test, n = 7.

Different from Porsolt’s forced swimming test, in MFST, durations of active movements such as swimming and climbing are taken into account in addition to the immobility behaviors of the animals. Evaluation of the active behaviors is critical, since it provides some additional clues related to the possible neurotransmitter systems playing role in the antidepressant-like effect of a test compound [45,46,47,48]. Namely, agents affecting serotonergic neurotransmission are known to reduce immobility and increase swimming times, in MFST. On the other hand, agents increasing catecholaminergic neurotransmission in the synaptic cleft have been reported to enhance climbing time while shortening the immobility durations of animals [47]. In the present study, effects of test compounds and fluoxetine on swimming and climbing time of mice in the MFST was demonstrated in Figure 3 [F(9,60) = 5.53, *p* < 0.001] and Figure 4 [F(9,60) = 0.82, *p* > 0.05], respectively. Results of the post-hoc analysis indicated that compounds **4a**, **4b**, **4e**, **4f** and fluoxetine significantly increased the swimming duration of mice while reducing the immobility time. On the other hand, none of the test compounds altered the climbing time of the animals. Therefore, it can be suggested that antidepressant-like effects of the compounds **4a**, **4b**, **4e** and **4f** might be related to the serotonergic rather than catecholaminergic mechanisms in the CNS.

**Figure 5:**
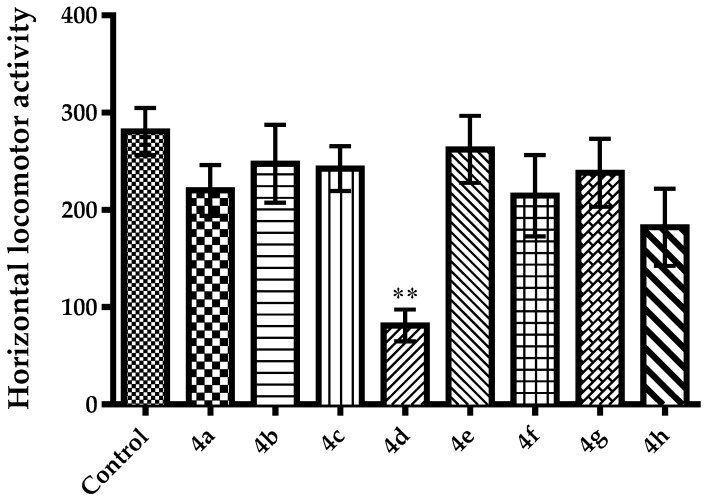
The effect of **4a**–**4h** (50 mg/kg) on horizontal locomotor activities of animals in the activity cage test. ** *p* < 0.01, compared with control group; One way analysis of variance followed by Tukey HSD multiple comparison test, n = 7.

**Figure 6:**
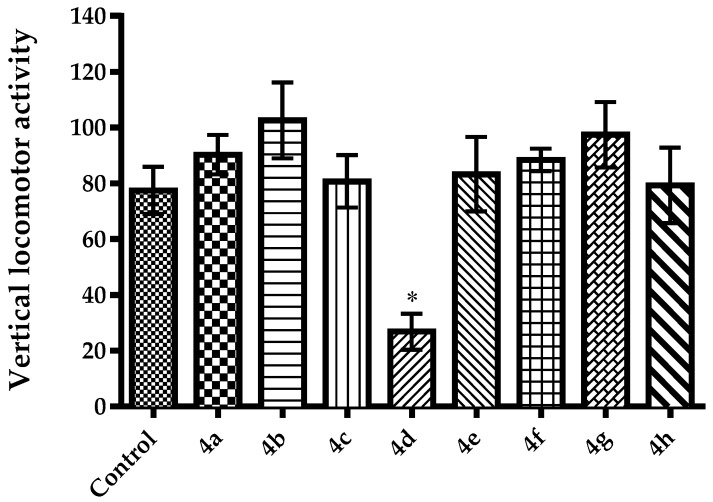
The effect of **4a**–**4h** (50 mg/kg) on vertical locomotor activities of animals in the activity cage test. * *p* < 0.05, compared with control group; One way analysis of variance followed by Tukey HSD multiple comparison test, n = 7.

It is known that agents altering motor activity performances of animals may cause “false positive” or “false negative” results, in TST and MFST [40,42,49]. For this reason, spontaneous locomotor activities of animals were also evaluated, in this study. Effects of the test compounds on horizontal and vertical locomotor activities of mice in the activity cage test were presented in Figure 5 [F(8,54) = 3.32, *p* < 0.01] and Figure 6 [F(8,54) = 4.52, *p* < 0.001], respectively. Compounds **4a**, **4b**, **4e** and **4f**, which show antidepressant-like activity in the TST and MFST, did not cause any significant changes in the horizontal and vertical spontaneous locomotor activities of the animals. These findings suggest that shortened immobility time of the animals in TST and MFST is not associated with stimulatory effects of compounds **4a**, **4b**, **4e** and **4f**. In other words, observed antidepressant-like effects are specific. On the other hand, compound **4d**, which did not cause any changes in immobility or swimming time of mice, triggered a significant reduction both in the horizontal and vertical locomotor activities of animals. These decreases in the spontaneous locomotor activities may be directly related to neurosedative activity of compound **4d** or possible effect of this compound on neuromuscular junction. Further studies are needed to clarify this issue. 

Following the antidepressant-like activity screening studies, we conducted some further mechanistic experiments. Instead of MFST, we conducted our studies by TST, because of the lack of hypothermia risk, enhanced pharmacological sensitivity, and rapid return to normal spontaneous activity following this test [40,50]. 

**Figure 7:**
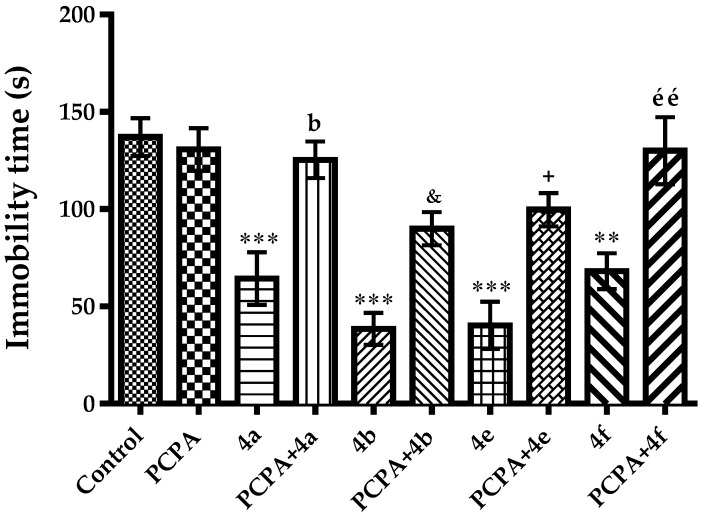
Effect of PCPA (100 mg/kg) pre-treatments on antidepressant-like activity induced by compounds **4a**, **4b**, **4e** and **4f** in the tail suspension test. ** *p* < 0.01, *** *p* < 0.001, compared with control group; ^b^ *p* < 0.01, compared with compound **4a** administrated group; ^&^ *p* < 0.05, compared with compound **4b** administrated group; ^+^ *p* < 0.05, compared with compound **4e** administrated group; ^éé^ *p* < 0.01, compared with compound **4f** administrated group; One way analysis of variance followed by Tukey HSD multiple comparison test, n = 7.

With the aim of examining the possible involvement of the serotonergic system to the antidepressant-like effects of the compounds **4a**, **4b**, **4e** and **4f**, we used p-chlorophenylalanine methyl ester (PCPA). PCPA is an agent inhibiting tryptophan hydroxylase enzyme playing role in the synthesis of serotonin. It stops the serotonin synthesis and reduces the endogenous serotonin storage by 60–90% in the CNS of mice, when administered at 100 mg/kg daily dose for four consecutive days. PCPA does not alter central noradrenaline or dopamine levels while reducing serotonin in brain [51,52,53]. In this study, effects of PCPA pre-treatments on anti-immobility activities of the compounds **4a**, **4b**, **4e** and **4f** in the TST were exhibited in Figure 7 [F(9,60) = 11.82, *p* < 0.001]. 

Pre-treatments with PCPA reversed the compounds **4a**, **4b**, **4e** and **4f** induced shortenings in the immobility time of mice. This data indicated that antidepressant-like effects of these compounds are associated with the serotonergic neurotransmission. This result is in accordance with our experimental data obtained from the MFST tests suggesting the involvement of serotonergic system in the observed antidepressant-like effects.

After realizing the importance of serotonergic neurotransmission in the pharmacological effects of the active compounds, we tried to clarify possible serotonergic receptors participating the mechanism of action. Therefore, probable contributions of 5-HT_1A_, 5-HT_2A/2C_ and 5-HT_3_ serotonergic receptors to the observed antidepressant-like effects were evaluated by pre-treatments with NAN-190, ketanserin and ondansetron, respectively [54,55]. 

**Figure 8:**
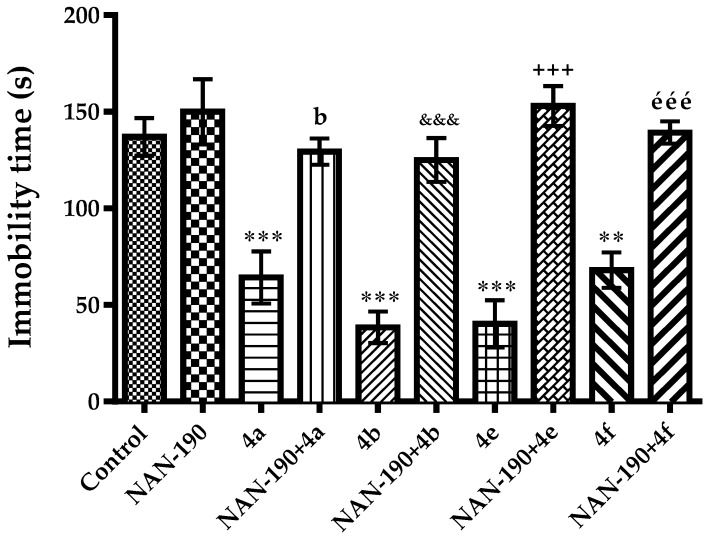
Effect of NAN-190 (0.5 mg/kg) pre-treatments on antidepressant-like activity induced by compounds **4a**, **4b**, **4e** and **4f** in the tail suspension test. ** *p* < 0.01, *** *p* < 0.001, compared with control group; ^b^ *p* < 0.01, compared with compound **4a** administrated group; ^&&&^ *p* < 0.001, compared with compound **4b** administrated group; ^+++^ *p* < 0.001, compared with compound **4e** administrated group; ^ééé^ *p* < 0.001, compared with compound **4f** administrated group; One way analysis of variance followed by Tukey HSD multiple comparison test, n = 7.

The effect of NAN-190 pre-treatment on anti-immobility activities of the compounds **4a**, **4b**, **4e** and **4f** are shown in Figure 8 [F(9,60) = 18.15, *p* < 0.001]. NAN-190 pre-treatments reversed the anti-immobility effects of these compounds pointing out the participation of 5-HT_1A_ receptors to the antidepressant-like effects induced by compounds **4a**, **4b**, **4e** and **4f**. 

**Figure 9:**
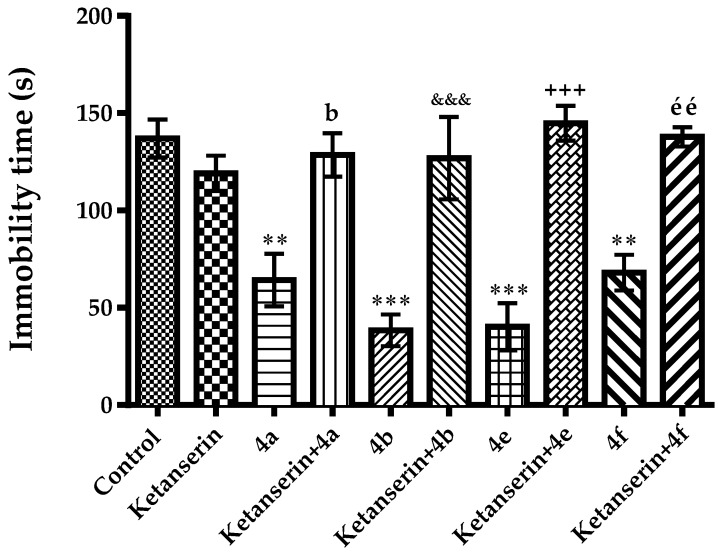
Effect of ketanserin (1 mg/kg) pre-treatments on antidepressant-like activity induced by compounds **4a**, **4b**, **4e** and **4f** in the tail suspension test. ** *p* < 0.01, *** *p* < 0.001, compared with control group; ^b^ *p* < 0.01, compared with compound **4a** administrated group; ^&&&^ *p* < 0.001, compared with compound **4b** administrated group; ^+++^ *p* < 0.001, compared with compound **4e** administrated group; ^éé^ *p* < 0.01, compared with compound **4f** administrated group; One way analysis of variance followed by Tukey HSD multiple comparison test, n = 7.

Figure 9 displays the effect of ketanserin pre-treatment on anti-immobility activities of the compounds **4a**, **4b**, **4e** and **4f** [F(9,60) = 13.66, *p* < 0.001]. 

Obtained data indicated that ketanserin pre-treatments antagonized the anti-immobility effects of these compounds pointing out the contribution of 5-HT_2A/2C_ receptors to the exhibited antidepressant-like effects. 

**Figure 10:**
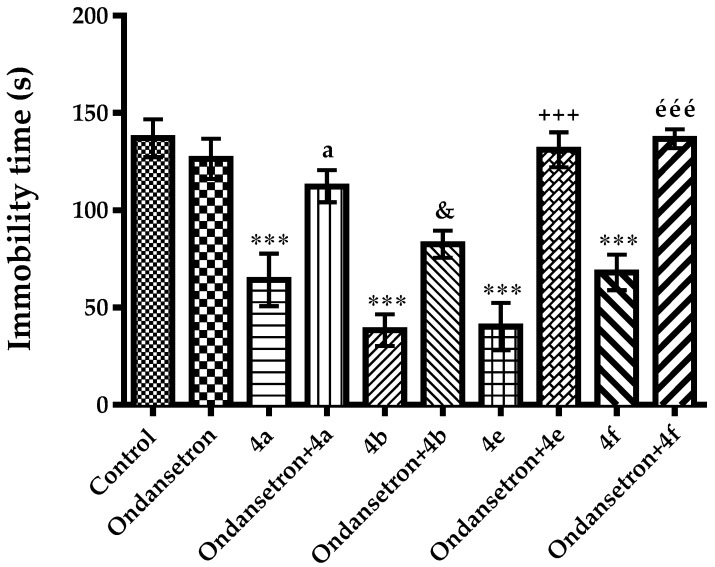
Effect of ondansetron (0.3 mg/kg) pre-treatments on antidepressant-like activity induced by compounds **4a**, **4b**, **4e** and **4f** in the tail suspension test. *** *p* < 0.001, compared with control group; ^a^ *p* < 0.05, compared with compound **4a** administrated group; ^&^ *p* < 0.05, compared with compound **4b** administrated group; ^+++^ *p* < 0.001, compared with compound **4e** administrated group; ^ééé^ *p* < 0.001, compared with compound **4f** administrated group; One way analysis of variance followed by Tukey HSD multiple comparison test, n = 7.

The effect of ondansetron pre-treatment on anti-immobility activities of the compounds **4a**, **4b**, **4e** and **4f** are shown in Figure 10 [F(9,60) = 17.36, *p* < 0.001]. Ondansetron pre-treatments reversed the anti-immobility effects induced by the compounds **4a**, **4b**, **4e** and **4f** suggesting the involvement of 5-HT_3_ receptors in the antidepressant-like effects of these compounds.

In summary, the findings acquired from the mechanistic studies indicated that compounds **4a**, **4b**, **4e** and **4f** presented significant antidepressant-like activities and 5-HT_1A_, 5-HT_2A/2C_ and 5-HT_3_ receptors seem to contribute to these effects. These results supported the previous literature pointing out the role of serotonergic system and 5-HT_1A_, 5-HT_2A/2C_ and 5-HT_3_ receptors [56,57,58,59] in the pharmacological mechanisms of various compounds possessing antidepressant efficacy. Actually, additional radioligand binding studies will help to confirm the involvement of these three serotonergic receptors as molecular targets of compounds **4a**, **4b**, **4e** and **4f**. On the other hand, other serotonergic receptor subtypes might also be participated to the observed pharmacological effect. Therefore, potential involvement of additional serotonergic receptors and even other neuromediator systems (GABAergic, glutaminergic, and nitrergic systems etc.) to the presented antidepressant-like activities should also be examined by further detailed studies. 

Results of the in vivo studies indicated that pharmacological activities of the compounds **4a**, **4b**, **4e** and **4f** (Figure 1, Figure 2 and Figure 3) are independent of whether they contain benzimidazole (compounds **4a**,**4b**) or benzothiazole (compounds **4e**,**4f**) rings on the chemical scaffold. Instead, substitutions at the 4^-^-position of the piperazine ring seem to cause the difference in the activity. More specifically, antidepressant-like effect was observed with the compounds bearing methyl and ethyl groups at the piperazine 4-position (compounds **4a**,**4b**,**4e** and **4f**), whereas isopropyl- and cyclopropyl-substituted compounds **4c**,**4d**,**4g** and **4h** were found to be ineffective. Therefore, it can be suggested that steric effects may play a role on the pharmacological activity, which is enhanced by smaller substituents such as methyl and ethyl, but abolished by relatively more bulky groups like isopropyl and cyclopropyl. On the other hand, it should be noted that increasing the number of test compounds in the series might provide a more reliable approach for the structure activity relationship related to the tested compounds.

## 3. Materials and Methods 

### 3.1. Chemistry

Chemicals of the Merck (Merck KGaA, Darmstadt, Germany) or Sigma-Aldrich (Sigma-Aldrich Corp., St. Louis, MO, USA) companies were used in the synthesis. The ^1^H-NMR and ^13^C-NMR spectra were recorded in DMSO-*d*_6_ at 300 MHz and 75 MHz, pespectively, on a digital FT-NMR spectrometer (Bruker Bioscience, Billerica, MA, USA). The NMR spectra signals were defined as a singlet (s), br.s. (broad singlet), doublet (d), triplet (t), double doublet (dd), and multiplet (m). The coupling constants (*J*) are reported in units of Hertz (Hz). A FT-IR spectrometer (IRAffinity-1S Fourier transform, Shimadzu, Tokyo, Japan) was used to record the IR spectra of the compounds. MS studies were performed on an LCMS-8040 tandem mass system (Shimadzu) and acetonitrile containing 0.1% formic acid was used as solvent. Silica gel 60 F254 by TLC (Merck KGaA) was used to monitor the progress of reactions.

#### 3.1.1. Synthesis of 4-Substituted Benzaldehydes **1a**–**1d**

A solution of 4-fluorobenzaldehyde (0.017 mol, 1.82 mL) and an appropriate secondary amine in DMF (10 mL) was refluxed (0.017 mol) in the presence of potassium carbonate (K_2_CO_3_, 0.017 mol, 2.35 g). After completion of reaction, the mixture was cooled with ice-water (10 mL) and extracted with ethyl acetate (3 × 20 mL). The ethyl acetate phases were combined, and the remaining product was collected after evaporation of the solvent. The following compounds were prepared in this way: *4-(4-methylpiperazin-1-yl)benzaldehyde* (**1a**), CAS No:27913-99-1, Yield 82%, m.p. = 55–57 °C (measured), m.p. = 58–60 °C (reported) [60]; *4-(4-ethylpiperazin-1-yl)benzaldehyde* (**1b**), CAS No:197638-76-9, Yield 79%, obtained as an oil; *4-(4-isopropylpiperazin-1-yl)benzaldehyde* (**1c**), CAS No:197638-78-1, Yield 84%, obtained as an oil and *4-(4-cylopropylpiperazin-1-yl)benzaldehyde* (**1d**), CAS No:1292778-23-4, Yield 78%, obtained as an oil.

#### 3.1.2. Synthesis of Ethyl 2-(Benzazol-2-yl-thio)acetates **2a**,**2b**

The corresponding benzazol-2-thiol (0.015 mol), ethyl 2-chloroacetate, (0.015 mol, 1.61 mL), and K_2_CO_3_ (0.015 mol, 2.07 g) were refluxed in acetone (70 mL) for 8–10 h. After completion of the reaction, the solvent was removed using reduced pressure. The precipitated product was washed with water, dried and recrystallized from EtOH. The following compounds were preapared in this way: *ethyl 2-(benzimidazole-2-ylthio)acetate* (**2a**), CAS No:5429-62-9, Yield 65%, m.p. = 99–101 °C (measured), m.p. = 97 °C (reported) [61]; *ethyl 2-(benzothiazol-2-ylthio)acetate* (**2b**), CAS No:24044-88-0, Yield 72%, m.p. = 45–47 °C (measured), m.p. = 42–44 °C (reported) [62].

#### 3.1.3. Synthesis of 2-((5-Substitutedbenzazol-2-yl)thio)acetohydrazides **3a**,**3b**

A solution (20 mL) of hydrazine hydrate (5 mL) in EtOH (15 mL) was added as portions into the reaction mixture of ethyl 2-((benzazol-2-yl)thio)acetate **2a**–**2b** (0.012 mol) in EtOH (50 mL). The reaction mixture was stirred for 5 h. The precipitated product was filtered, washed with cold-ethanol, dried and recrystallized from EtOH. To afford the following compounds: *2-((1H-benzimidazol-2-yl)thio)acetohydrazide* (**3a**), CAS No:30065-27-1, Yield 62%, m.p. = 164–166 °C (measured), m.p. = 165–166 °C (reported) [63]; *2-((1H-benzothiazole-2-yl)thio)acetohydrazide* (**3b**), CAS No:24044-91-5, Yield 81%, m.p. = 175–177 °C (measured), m.p. = 173–174 °C (reported) [62]. 

#### 3.1.4. General Procedure for the Synthesis of the Target Compounds **4a**–**4h**

An appropriate 2-((benzazol-2-yl)thio) acetohydrazide **3a**–**3b** (0.002 mol), and a 4-substituted-benzaldehyde **1a**–**1h** (0.002 mol) and a catalytic amount of acetic acid were refluxed in EtOH (30 mL) for 2 h. The mixture was cooled in an ice-bath, and the precipitated product was filtered, dried and recrystallized from EtOH.

*2-((1H-Benzimidazol-2-yl)thio)-N′-(4-(4-methylpiperazin-1-yl)benzylidene)acetohydrazide* (**4a**). Yield: 88%, FTIR (ATR, cm^−1^): 3572 (N-H), 2937 (C-H), 1660 (C=O), 817. ^1^H-NMR (DMSO-*d*_6_): δ = 2.22 (3H, s, -CH_3_), 2.42–2.45 (4H, m, piperazine), 3.21–3.24 (4H, m, piperazine), 3.37 (2H, s, -CH_2_), 6.98 (2H, d, *J* = 8.85 Hz, Ar-H), 7.07 (1H, t, *J* = 7.80 Hz, Ar-H), 7.19 (1H, t, *J* = 7.44 Hz, Ar-H), 7.32–7.37 (1H, m, Ar-H), 7.48–7.56 (3H, m, Ar-H), 8.07 (1H, s, Ar-H), 11.56 (1H, br.s, -NH).^13^C-NMR (DMSO-*d*_6_): δ = 40.82, 46.20, 47.65, 54.90, 109.68, 110.66, 115.04, 116.72, 121.49, 124.52, 128.31, 145.45, 152.24, 159.99. ESI-MS [M + H]^+^: 409.00 (100%).

*2-((1H-Benzimidazol-2-yl)thio)-N′-(4-(4-ethylpiperazin-1-yl)benzylidene)acetohydrazide* (**4b**). Yield: 82%, FTIR (ATR, cm^−1^): 3562 (N-H), 2970 (C-H), 1660 (C=O), 817. ^1^H-NMR (DMSO-*d*_6_): δ = 1.03 (3H, t, *J* = 7.17 Hz, -CH_3_), 2.36 (2H, q, *J* = 7.20 Hz, -CH_2_-) 2.47–2.50 (4H, m, piperazine), 3.21–3.24 (4H, m, piperazine), 3.35 (2H, s, -CH_2_), 6.98 (2H, d, *J* = 8.85 Hz, Ar-H), 7.07 (1H, t, *J* = 7.74 Hz, Ar-H), 7.19 (1H, t, *J* = 7.50 Hz, Ar-H), 7.35–7.37 (1H, m, Ar-H), 7.49–7.56 (3H, m, Ar-H), 8.07 (1H, s, Ar-H), 11.80 (1H, br.s, -NH). ^13^C-NMR (DMSO-*d*_6_): δ = 12.45, 40.89, 47.76, 52.08, 52.67, 109.67, 114.99, 116.71, 121.48, 124.52, 128.29, 142.94, 145.47, 148.55, 152.27, 160.00. ESI-MS [M + H]^+^: 423.10 (100%).

*2-((1H-Benzimidazol-2-yl)thio)-N′-(4-(4-isopropylpiperazin-1-yl)benzylidene)acetohydrazide* (**4c**). Yield: 80%, FTIR (ATR, cm^−1^): 3562 (N-H), 2966 (C-H), 1708 (C=O), 806. ^1^H-NMR (DMSO-*d*_6_): δ = 0.99 (6H, d, *J* = 6.45 Hz, -CH_3_), 2.55 (4H, br.s., piperazine), 2.62–2.69 (1H, m, -CH-), 3.21 (4H, br.s., piperazine), 4.64 (2H, s, -CH_2_-), 6.95 (2H, d, *J* = 8.85 Hz, Ar-H), 7.31–7.34 (2H, m, Ar-H), 7.53 (2H, d, *J* = 8.76 Hz, Ar-H), 7.63–7.66 (2H, m, Ar-H), 7.93 (1H, s, Ar-H), 11.58 (1H, s, -NH). ^13^C-NMR (DMSO-*d*_6_): δ = 18.67, 35.26, 48.08, 48.38, 54.12, 110.67, 114.90, 118.72, 124.04, 124.71, 125.09, 128.58, 141.73, 144.94, 148.04, 152.61, 162.74, 167.97. ESI-MS [M + H]^+^: 437.20 (100%).

*2-((1H-Benzimidazol-2-yl)thio)-N′-(4-(4-cyclopropylpiperazin-1-yl)benzylidene)acetohydrazide* (**4d**). Yield: 79%, FTIR (ATR, cm^−1^): 3550 (N-H), 2974 (C-H), 1718 (C=O), 810. ^1^H-NMR (DMSO-*d*_6_): δ = 0.34–0.35 (2H, m, -CH_2_-), 0.42-0.44 (2H, m, -CH_2_-), 1.62-1.65 (1H, m, -CH-), 2.65 (4H, br.s., piperazine), 3.17–3.20 (4H, m, piperazine), 4.65 (2H, s, -CH_2_-), 6.93–6.98 (2H, m, Ar-H), 7.07 (1H, t, *J* = 7.86 Hz, Ar-H), 7.19 (1H, t, *J* = 6.81, Ar-H) 7.31–7.33 (1H, m), 7.51–7.57 (2H, m, Ar-H), 7.63–7.65 (1H, m, Ar-H), 11.59 (1H, s, -NH). ^13^C-NMR (DMSO-*d*_6_): δ = 6.09, 35.27, 38.52, 47.78, 52.99, 53.04, 110.66, 115.07, 118.72, 121.46, 124.71, 125.09, 128.58, 141.73, 144.92, 148.01, 152.25, 162.76, 167.97. ESI-MS [M + H]^+^: 435.20 (100%).

*2-(Benzothiazol-2-ylthio)-N′-(4-(4-methylpiperazin-1-yl)benzylidene)acetohydrazide* (**4e**). Yield: 81%, FTIR (ATR, cm^−1^): 3313 (N-H), 2933 (C-H), 1660 (C=O), 819. ^1^H-NMR (DMSO-*d*_6_): δ = 2.22 (3H, s, -CH_3_), 2.42–2.43 (4H, m, piperazine), 3.21–3.24 (4H, m, piperazine), 4.66 (2H, s, -CH_2_), 6.96 (2H, t, *J* = 9.63 Hz, Ar-H), 7.34–7.40 (1H, m, Ar-H), 7.44–7.55 (3H, m, Ar-H), 7.85 (1H, d, *J* = 8.07 Hz, Ar-H), 7.93 (1H, s, Ar-H), 8.00–8.04 (1H, m, Ar-H), 11.54 (1H, s, -NH). ^13^C-NMR (DMSO-*d*_6_): δ = 35.61, 46.20, 47.57, 54.85, 114.98, 121.58, 122.30, 124.17, 124.92, 126.84, 128.56, 135.20, 144.74, 147.99, 152.49, 162.98, 168.29. ESI-MS [M + H]^+^: 426.10 (100%).

*2-(Benzothiazol-2-ylthio)-N′-(4-(4-ethylpiperazin-1-yl)benzylidene)acetohydrazide* (**4f**). Yield: 82%, FTIR (ATR, cm^−1^): 3228 (N-H), 2966 (C-H), 1662 (C=O), 821. ^1^H-NMR (DMSO-*d*_6_): δ = 1.02 (3H, t, *J* = 6.63 Hz, -CH_3_), 2.34 (2H, q, *J* = 7.32 Hz), 2.46 (4H, br.s., piperazine), 3.21 (4H, br.s., piperazine), 4.64 (2H, s, -CH_2_-), 6.94 (2H, d, *J* = 8.73 Hz, Ar-H), 7.35 (1H, m, Ar-H), 7.43–7.51 (3H, m, Ar-H), 7.84 (1H, d, *J* = 7.92 Hz, Ar-H), 7.91 (1H, s, Ar-H), 8.01 (1H, d, *J* = 8.67 Hz, Ar-H), 11.52 (1H, s, -NH). ^13^C-NMR (DMSO-*d*_6_): δ = 17.18, 40.35, 52.43, 56.82, 57.37, 119.68, 126.33, 127.05, 128.91, 129.67, 131.60, 133.31, 139.95, 149.50, 152.75, 157.27, 167.73, 173.04. ESI-MS [M + H]^+^: 440.11 (100%).

*2-(Benzothiazol-2-ylthio)-N′-(4-(4-isopropylpiperazin-1-yl)benzylidene)acetohydrazide* (**4g**). Yield: 82%, FTIR (ATR, cm^−1^): 3471 (N-H), 2960 (C-H), 1668 (C=O), 833. ^1^H-NMR (DMSO-*d*_6_): δ = 0.99 (6H, d, *J* = 6.51 Hz, -CH_3_), 2.50-2.51 (1H, m, -CH-), 2.54–2.57 (4H, m, piperazine), 3.19–3.22 (4H, m, piperazine), 4.66 (2H, s, -CH_2_-), 6.94 (2H, t, *J* = 9.67 Hz, Ar-H), 7.34–7.40 (1H, m, Ar-H), 7.44–7.47 (1H, m, Ar-H), 7.51 (1H, d, *J* = 6.30 Hz, Ar-H), 7.54 (1H, d, *J* = 6.12 Hz, Ar-H), 7.84–7.89 (1H, m, Ar-H), 7.93 (1H, s, Ar-H), 8.00–8.04 (1H, m, Ar-H), 11.53 (1H, s, -NH). ^13^C-NMR (DMSO-*d*_6_): δ = 18.66, 35.60, 48.07, 48.37, 54.13, 114.89, 121.58, 122.30, 124.09, 124.92, 126.84, 128.55, 128.85, 135.20, 144.76, 148.02, 152.57, 162.97, 168.29. ESI-MS [M + H]^+^: 454.13 (100%).

*2-(Benzothiazol-2-ylthio)-N′-(4-(4-cyclopropylpiperazin-1-yl)benzylidene)acetohydrazide* (**4h**). Yield: 82%, FTIR (ATR, cm^−1^): 3228 (N-H), 2821 (C-H), 1654 (C=O), 821. ^1^H-NMR (DMSO-*d*_6_): δ = 0.34–0.36 (2H, m, -CH_2_-), 0.41–0.44 (2H, m, -CH_2_-), 1.62–1.66 (1H, m, -CH-), 2.63–2.65 (4H, m, piperazine), 3.16–3.19 (4H, m, piperazine), 4.65 (2H, s, -CH_2_-), 6.94 (2H, t, *J* = 9.46 Hz, Ar-H), 7.34-7.39 (1H, m, Ar-H), 7.44–7.47 (1H, m, Ar-H), 7.50 (1H, d, *J* = 6.24 Hz, Ar-H), 7.53 (1H, d, *J* = 5.94 Hz, Ar-H), 7.85 (1H, d, *J* = 8.10 Hz, Ar-H), 7.92 (1H, s, Ar-H), 7.99–8.03 (1H, m, Ar-H), 11.53 (1H, s, -NH). ^13^C-NMR (DMSO-*d*_6_): δ = 6.08, 35.61, 47.61, 47.70, 52.99, 115.01, 121.58, 122.29, 124.16, 124.92, 126.84, 128.56, 135.20, 144.73, 147.98, 152.51, 162.97, 168.29. ESI-MS [M + H]^+^: 452.12 (100%).

### 3.2. Pharmacology

#### 3.2.1. Animals

Adult Balb/c mice weighing 30–35 g were used for the behavioral tests. They were group-housed and maintained in standard laboratory conditions: constant temperature (24 ± 1 °C) and 12-h light/dark cycle. Animals were allowed to acclimatize to the laboratory conditions 48 h before the experiments. The experimental protocol of this study was approved by the Local Ethical Committee on Animal Experimentation of Anadolu University (Eskişehir, Turkey; date: 13.04.2018; Project no: 2018-21).

#### 3.2.2. Experimental Groups and Treatments

Animals were randomly placed into 10 groups with seven animals in each. Efficacies of the test compounds (50 mg/kg, po) [21] were compared to control and fluoxetine (reference drug, 20 mg/kg, po) groups [37]. Vehicle, fluoxetine and test compounds were administered three times to mice at 24, 5 and 1.0 h before the experiments [37,64].

#### 3.2.3. Evaluation of Antidepressant-Like Activity

##### Tail Suspension Test

TST was carried out as previously described [36,41]. In this test, mice were suspended 30 cm above the ground with an adhesive tape, which is located about one cm from tip of the tails. Animals were evaluated as immobile when they did not perform escape-oriented movements and become motionless. Total duration of the immobility behavior was measured by a chronometer over the last 4 min of the 6-min test period. Mice climbing their tails during the tests were eliminated from the tests.

##### Modified Forced Swimming Test

MFST was also performed to examine the antidepressant-like effects of test compounds. In this test, mice were forced to swim in a Plexiglas cylinder (30 cm height, 12 cm diameter) filled with water up to 20 cm high. The temperature of the water was set at 25 ± 1 °C. Twenty-four hours before the experiments, animals were let to swim individually in the cylinder for 15 min. In the experimental phase, mice were placed again in the cylinder and durations of immobility (the movements required to keep the head above the water), swimming (horizontal motion on the surface of the water) and climbing (upward-directed movements of the forepaws along the side of the swim chamber) behaviors over 5-s intervals were measured with a chronometer for 5 min [36,47,65]. 

The water in the cylinder was changed after each mouse. After pre-test and experimental phases, mice were dried carefully before returning their home cages.

#### 3.2.4. Evaluation of Spontaneous Locomotor Activity

##### Activity Cage Test

Activity cage device (No. 7420, Ugo Basile, Varese, Italy) was used to assess the effects of test compounds on motor activities of mice. In this device, numbers of movements interrupting the IR beams are recorded, automatically. Horizontal and vertical spontaneous locomotor activities were recorded for 4 min. After testing each animal, the apparatus was carefully cleaned with ethanol solution [37,66]. 

##### Mechanistic Studies

Mechanistic studies were performed with PCPA (a serotonin synthesis inhibitor), NAN-190 (5-HT_1A_ receptor antagonist), ketanserin (5-HT_2A/2C_ receptor antagonist) and ondansetron (5-HT_3_ receptor antagonist) to investigate the possible involvement of the serotonergic system and serotonergic receptors in the antidepressant-like effects of test compounds. PCPA was intraperitoneally administered at dose of 100 mg/kg for four consecutive days. Test compounds were applied 24 h after the last injection and 1 h later, animals were subjected to the TST [54]. NAN-190 (0.5 mg/kg i.p.), ketanserin (1 mg/kg i.p.), ondansetron (0.3 mg/kg i.p.) were injected 15 min prior to the administration of the test compounds. Then, TST was conducted 1 h later [36]. 

##### Statistical Analysis

Graphpad Prism ver. 6.01 (GraphPad Software, San Diego, CA, USA) was used for statistical analyses and for the preparation of graphical presentations. Obtained data was analyzed using one-way analysis of variance (ANOVA) followed by Tukey’s multiple comparisons test. Results were expressed as the mean ± standard error of the mean (SEM). Differences between the groups were considered as statistically significant if *p* < 0.05.

## 4. Conclusions

Data obtained from this study presented that test compounds **4a**, **4b**, **4e** and **4f** possess significant antidepressant-like efficacy mediated through serotonergic system and 5-HT_1A_, 5-HT_2A/2C_ 5-HT_3_ serotonergic receptors. These results supported to the previous literature findings pointing out the antidepressant capacity of benzazole derivative compounds [20,21,22,26,27]. On the other hand, to clarify the exact mechanism of action possible contribution of other neurotransmitter systems (such as opioidergic, GABAergic, glutaminergic, and nitrergic systems) should also be investigated by further studies. 

## Figures and Tables

**Table:** 

**Comp.**	**X**	**R**
**4a**	-NH	Methyl
**4b**	-NH	Ethyl
**4c**	-NH	Isopropyl
**4d**	-NH	Cyclopropyl
**4e**	-S	Methyl
**4f**	-S	Ethyl
**4g**	-S	Isopropyl
**4h**	-S	Cyclopropyl

## Supplementary Materials

The ^13^C-NMR, ^1^H-NMR, FTIR, and MS spectrums of compounds **4a**–**4h** are available online.
