# Supplementary material for: Synthesis of Novel Benzazole Derivatives and Evaluation of Their Antidepressant-Like Activities with Possible Underlying Mechanisms
Source: Molecules. 2018 Nov 5;23(11):2881. doi: 10.3390/molecules23112881 (PMC6278502; doi:10.3390/molecules23112881)
Supplement: Supplementary file 1 [file molecules-23-02881-s001.pdf]

DOPNALAB

	Item	Value
	Acquired Date&Time	13.04.2018 15:38:48
	Acquired by	System Administrator
	Filename	C:\Users\dopnalab\Desktop\derya\GAMZE TOKGÖZ\Bİ-ME1.ispd
	Spectrum name	Bİ-ME1
	Sample name	Bİ-ME
	Sample ID	
	Option	
	Comment	
	No. of Scans	10
	Resolution	4 [cm-1]
	Apodization	Happ-Genzel

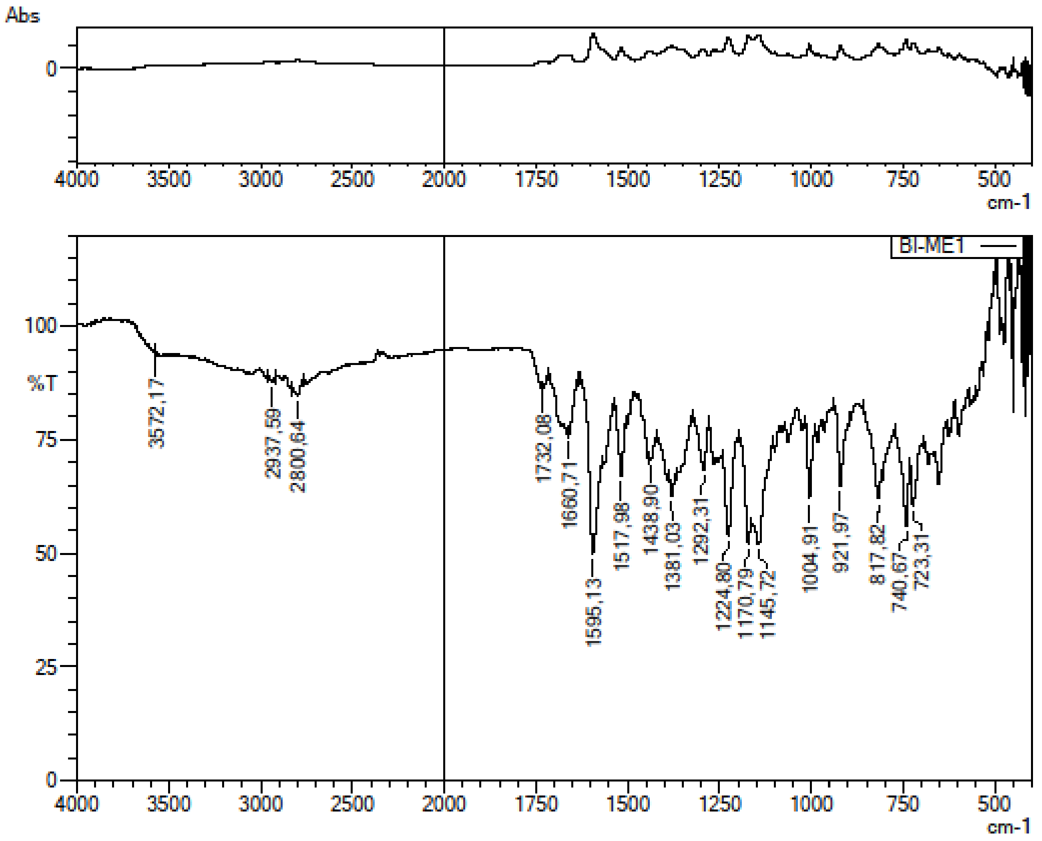

Spectra 1. IR spectra of compound **4a**

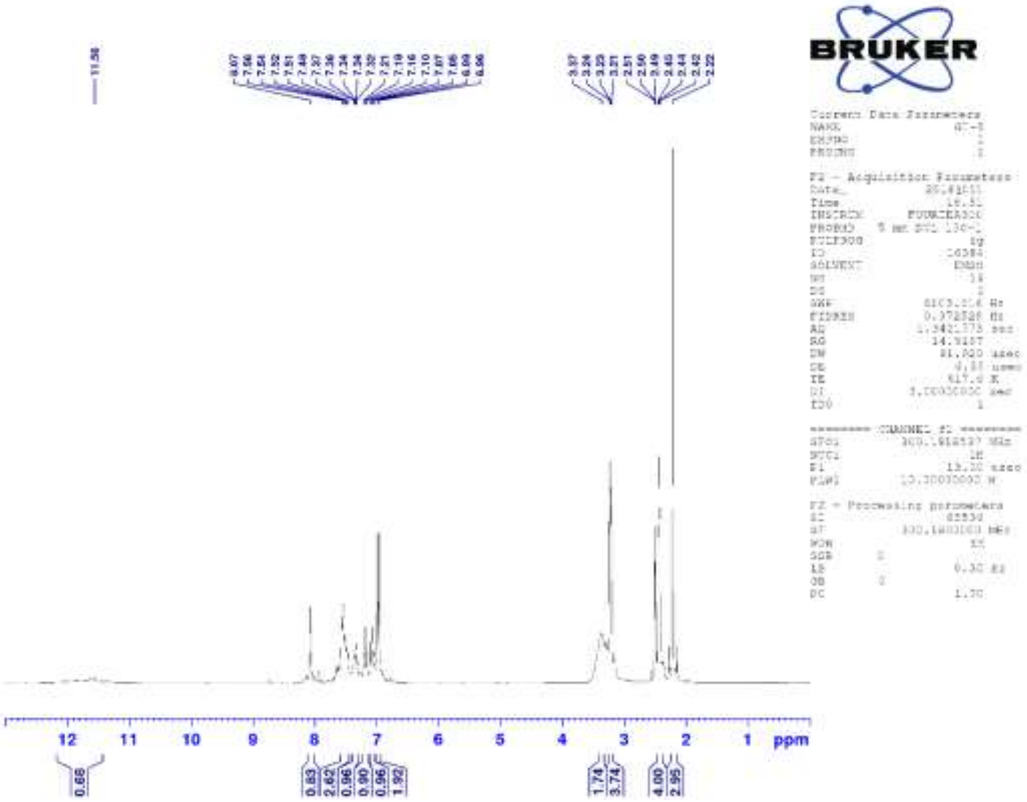

Spectra 2. ^{1}H-NMR spectra of compound **4a**

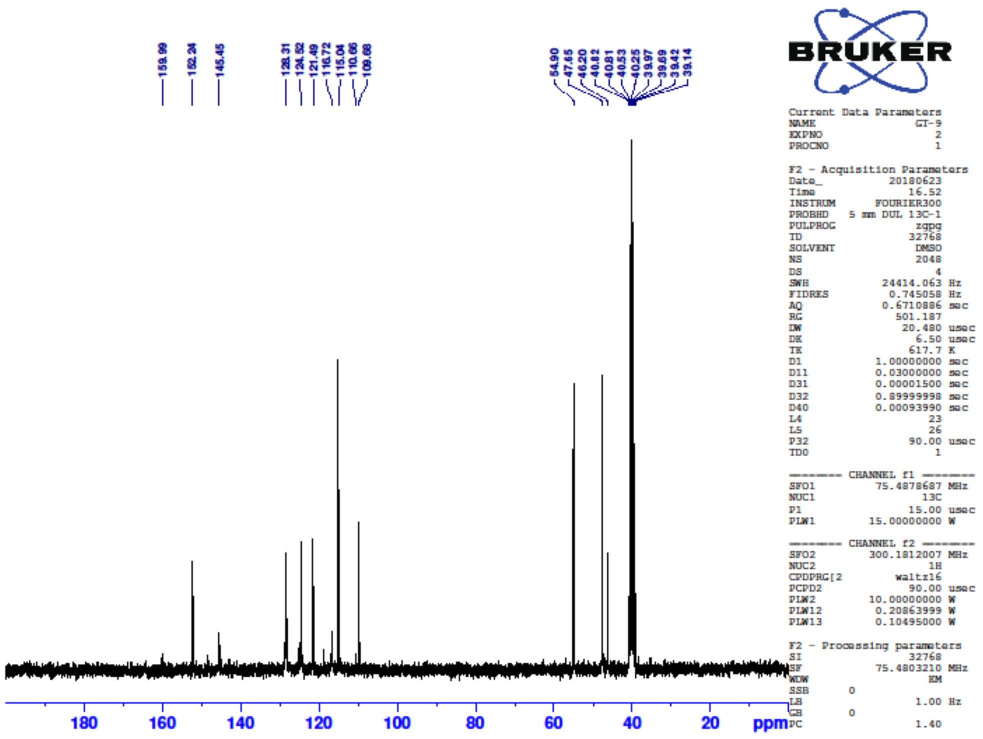

Spectra 3. ^{13}C-NMR spectra of compound **4a**

29.06.2018 16:22:40 Page 1 / 1

SHIMADZU LabSolutions

Analysis Report

<Sample Information>

Sample Name	: BI-Me		
Sample ID	:		
Data Filename	: BI-Me__004.lcd		
Method Filename	: genel.lcm		
Batch Filename	: batch.lcb		
Vial #	: 1-4	Sample Type	: Unknown
Injection Volume	: 0,1 uL		
Date Acquired	: 29.06.2018 16:18:41	Acquired by	: System Administrator
Date Processed	: 29.06.2018 16:19:44	Processed by	: System Administrator

<Chromatogram>

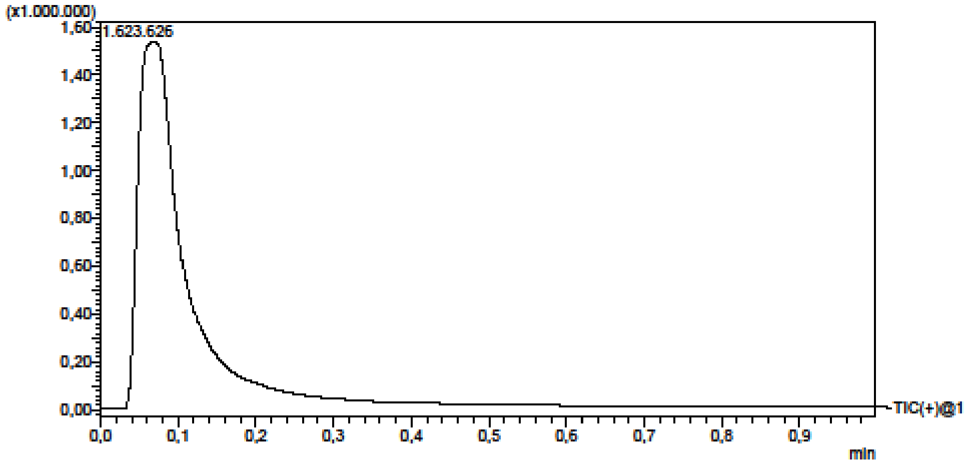

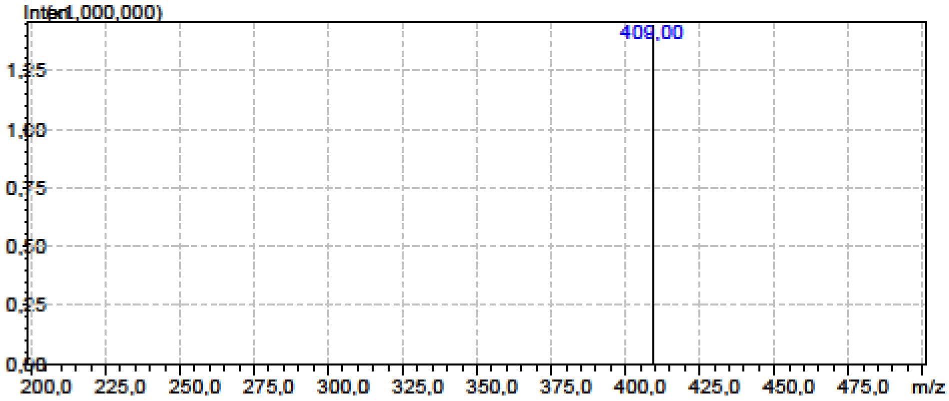

C:\LabSolutions\Data\serkan\Yeni klasör\BI-Me__004.lcd

Spectra 4. LCMSMS spectra of compound **4a**

DOPNALAB

	Item	Value
	Acquired Date&Time	13.04.2018 15:42:19
	Acquired by	System Administrator
	Filename	C:\Users\dopnalab\Desktop\derya\GAMZE TOKGÖZ\BI-ET1.ispd
	Spectrum name	BI-ET1
	Sample name	BI-ET
	Sample ID	
	Option	
	Comment	
	No. of Scans	10
	Resolution	4 [cm-1]
	Apodization	Happ-Genzel

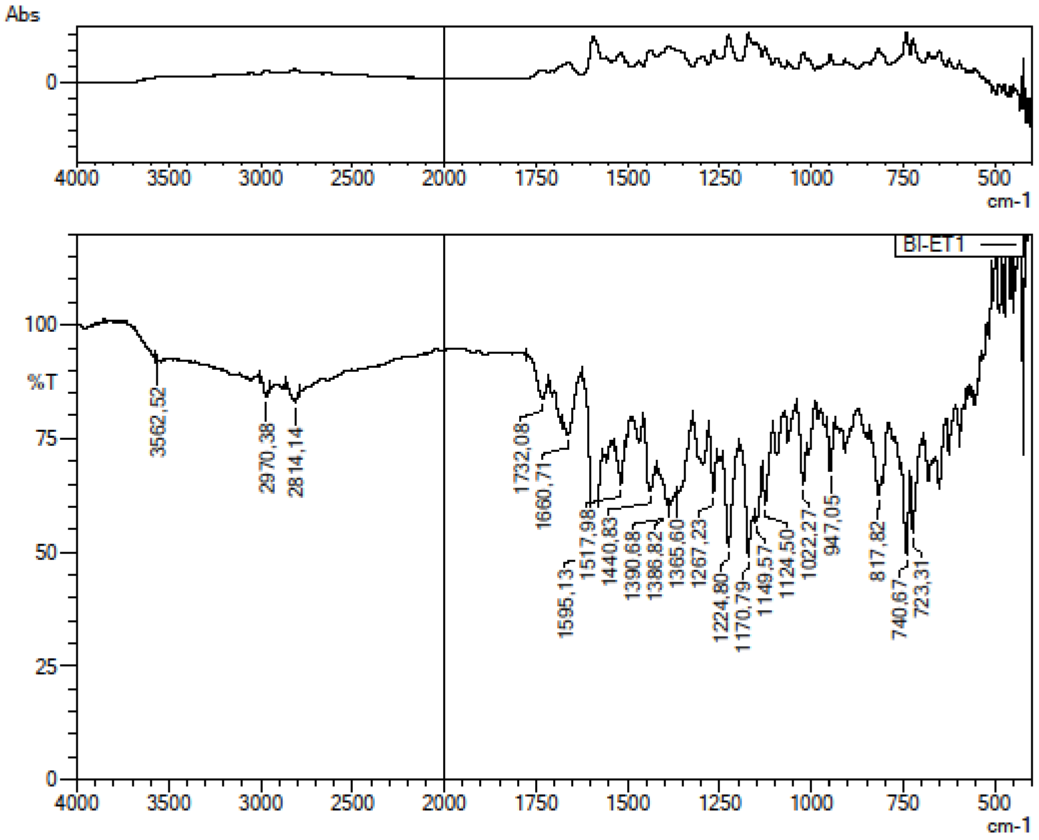

Spectra 5. IR spectra of compound **4b**

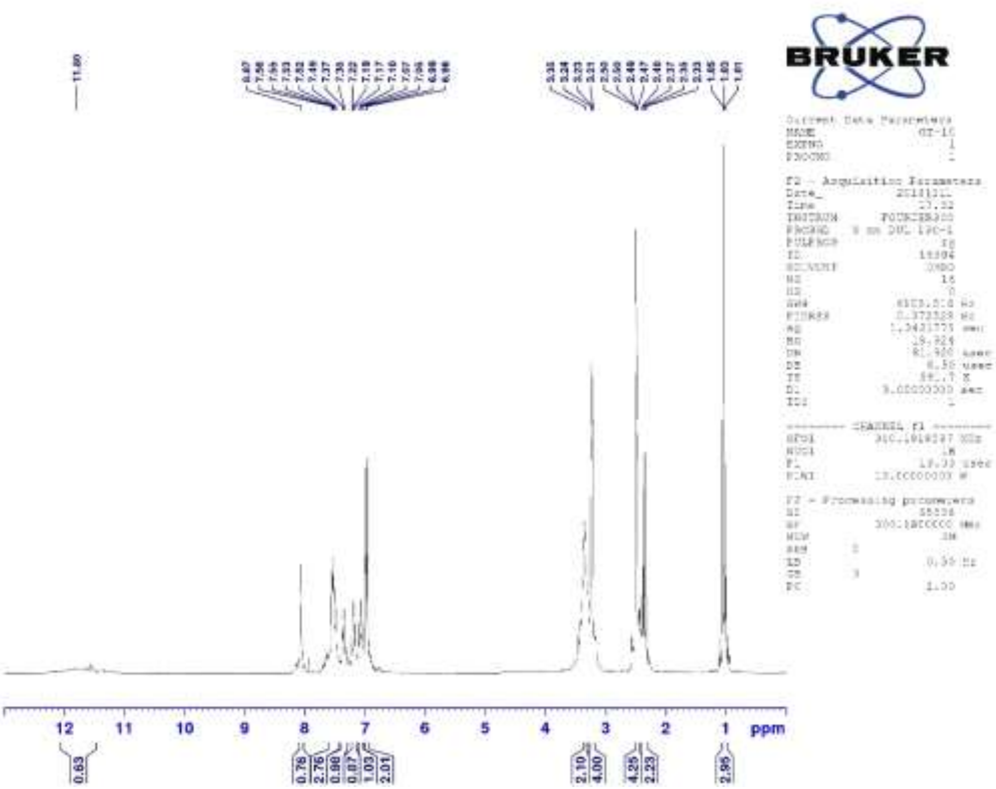

Spectra 6. ^{1}H-NMR spectra of compound **4b**

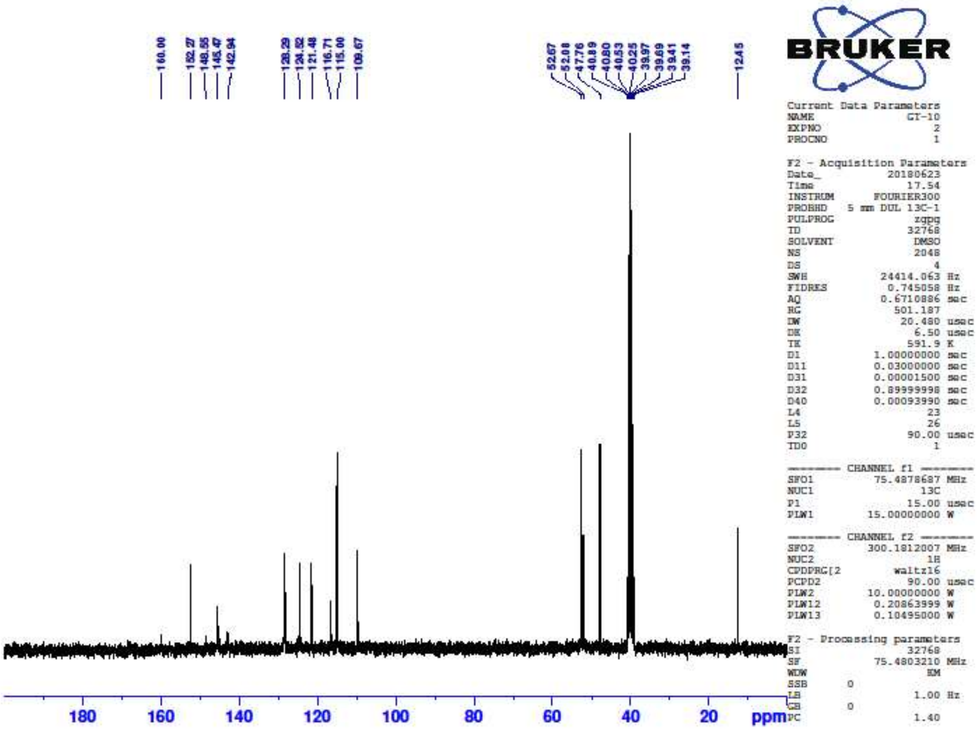

Spectra 7. ^{13}C-NMR spectra of compound **4b**

SHIMADZU LabSolutions

Analysis Report

<Sample Information>

Sample Name : Bl-et
Sample ID :
Data Filename : Bl-et__001.lcd
Method Filename : genel.lcm
Batch Filename : batch.lcb
Vial # : 1-5
Injection Volume : 0,1 uL
Date Acquired : 29.06.2018 16:26:55
Date Processed : 29.06.2018 16:27:59

Sample Type : Unknown
Acquired by : System Administrator
Processed by : System Administrator

<Chromatogram>

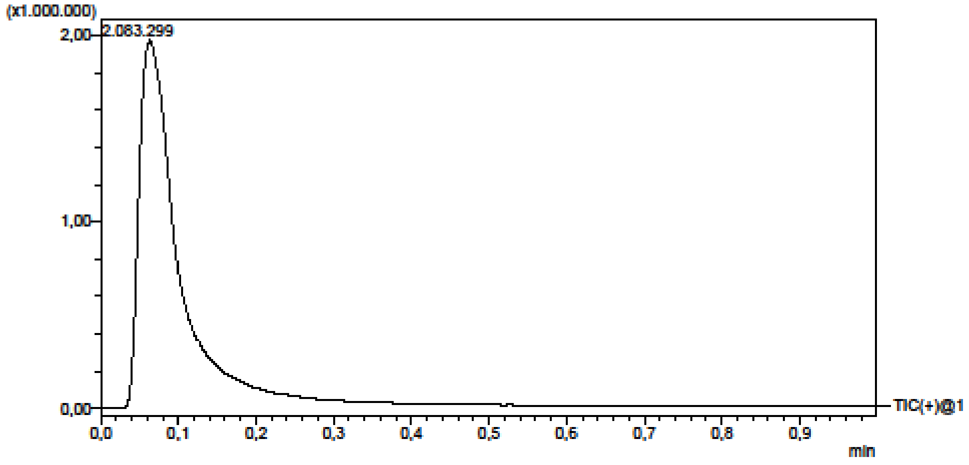

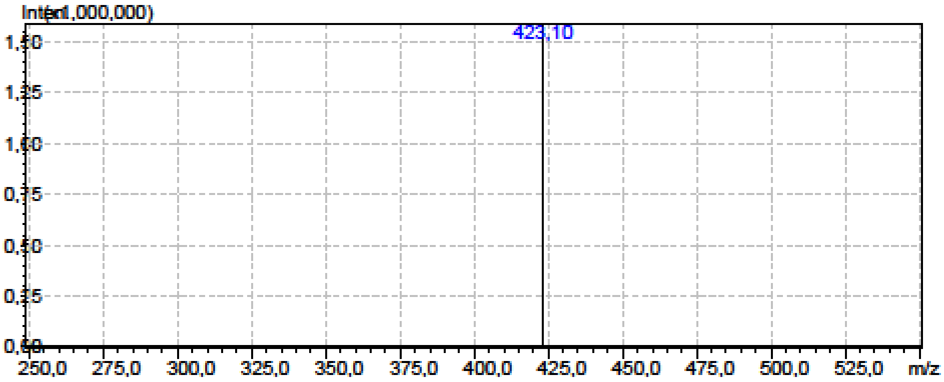

Spectra 8. LCMSMS spectra of compound **4b**

DOPNALAB

Item	Value
Acquired Date&Time	13.04.2018 15:49:00
Acquired by	System Administrator
Filename	C:\Users\dopnalab\Desktop\derya\GAMZE TOKGÖZ\BI-ISO1.ispd
Spectrum name	BI-ISO1
Sample name	BI-ISO
Sample ID	
Option	
Comment	
No. of Scans	10
Resolution	4 [cm-1]
Apodization	Happ-Genzel

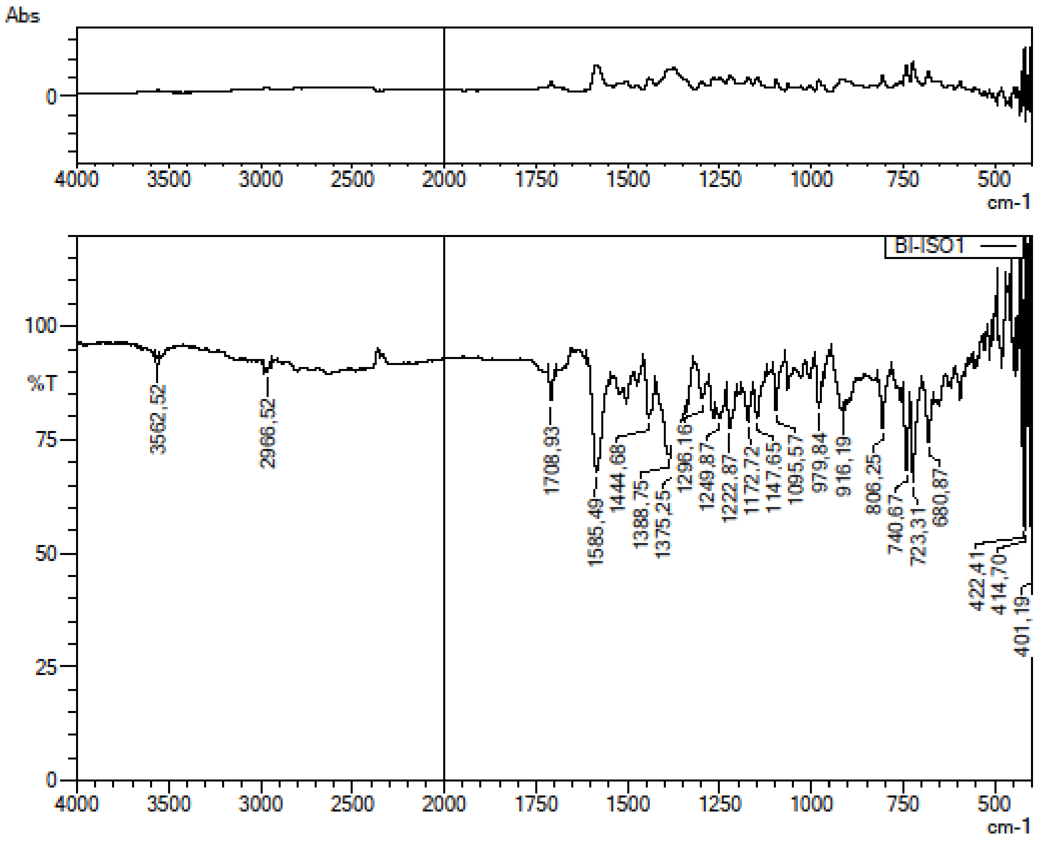

Spectra 9. IR spectra of compound **4c**

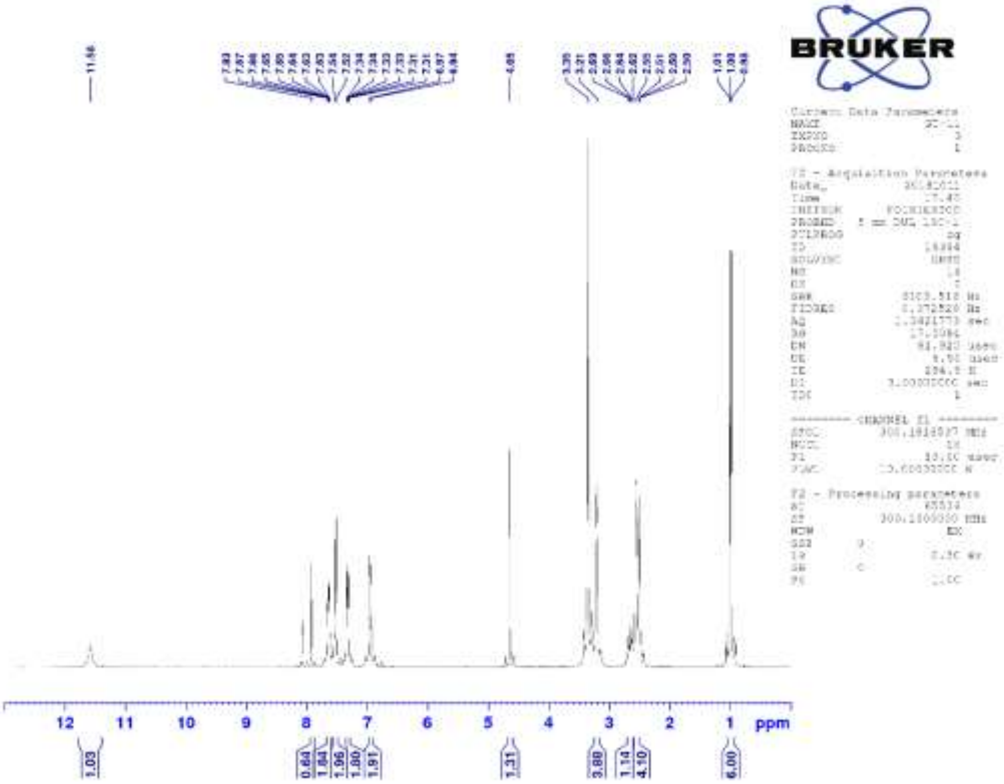

Spectra 10. ^{1}H-NMR spectra of compound **4c**

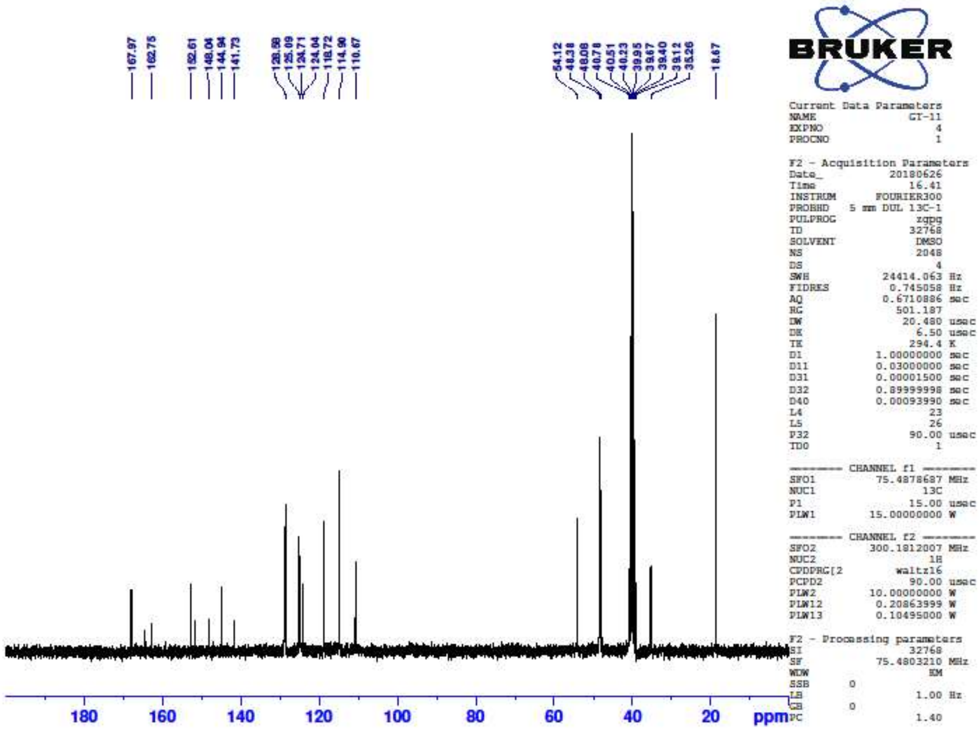

Spectra 11. ^{13}C-NMR spectra of compound **4c**

SHIMADZU LabSolutions

Analysis Report

<Sample Information>

Sample Name	: Bl-iso		
Sample ID	:		
Data Filename	: Bl-iso__001.lcd		
Method Filename	: genel.lcm		
Batch Filename	: batch.lcb		
Vial #	: 1-6	Sample Type	: Unknown
Injection Volume	: 0,1 uL		
Date Acquired	: 29.06.2018 16:31:40	Acquired by	: System Administrator
Date Processed	: 29.06.2018 16:32:43	Processed by	: System Administrator

<Chromatogram>

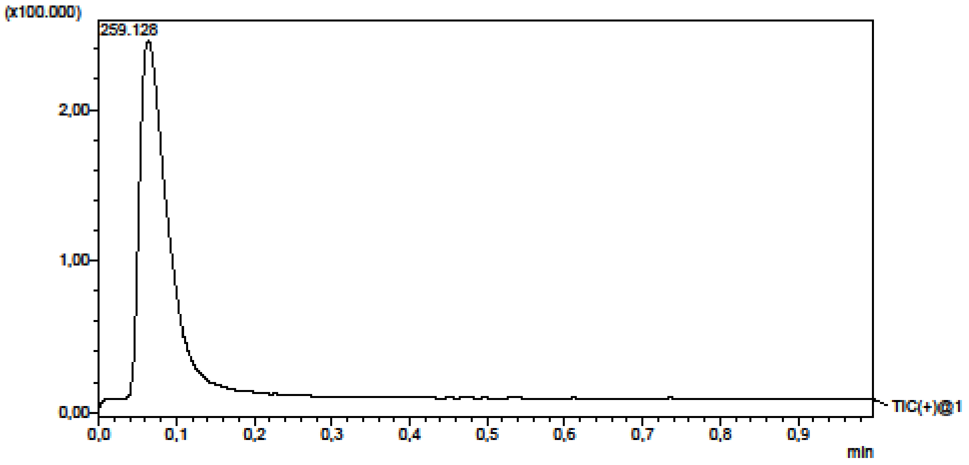

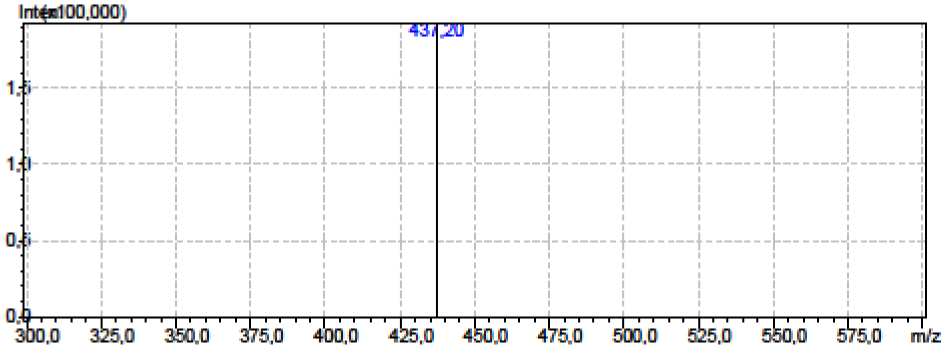

Spectra 12. LCMSMS spectra of compound **4c**

DOPNALAB

	Item	Value
	Acquired Date&Time	13.04.2018 15:53:03
	Acquired by	System Administrator
	Filename	C:\Users\dopnalab\Desktop\derya\GAMZE TOKGÖZ\BI-CYL1.ispd
	Spectrum name	BI-CYL1
	Sample name	BI-CYL
	Sample ID	
	Option	
	Comment	
	No. of Scans	10
	Resolution	4 [cm-1]
	Apodization	Happ-Genzel

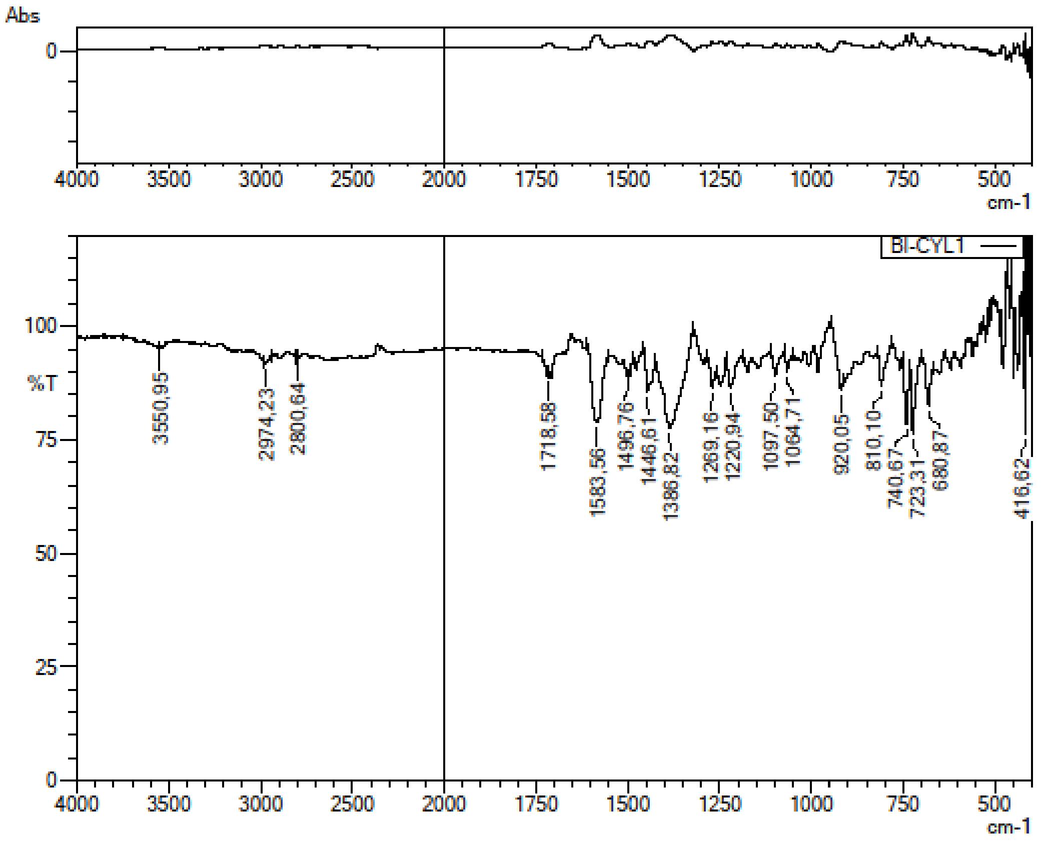

Spectra 13. IR spectra of compound **4d**

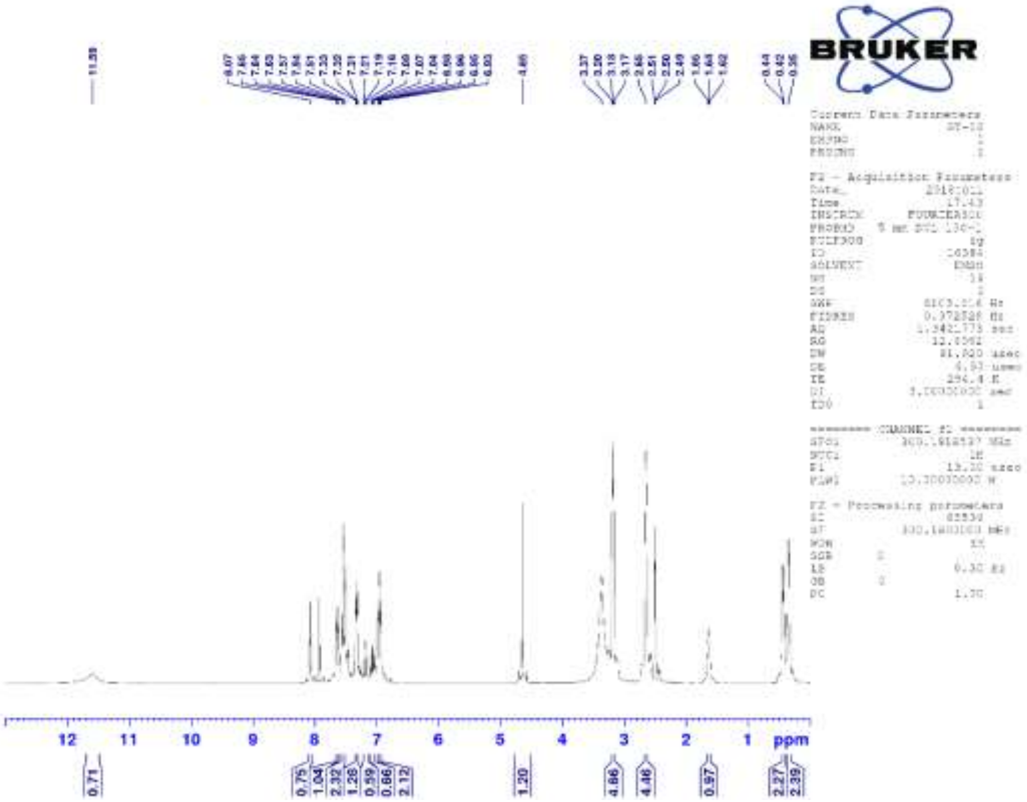

Spectra 14. ^{1}H-NMR spectra of compound **4d**

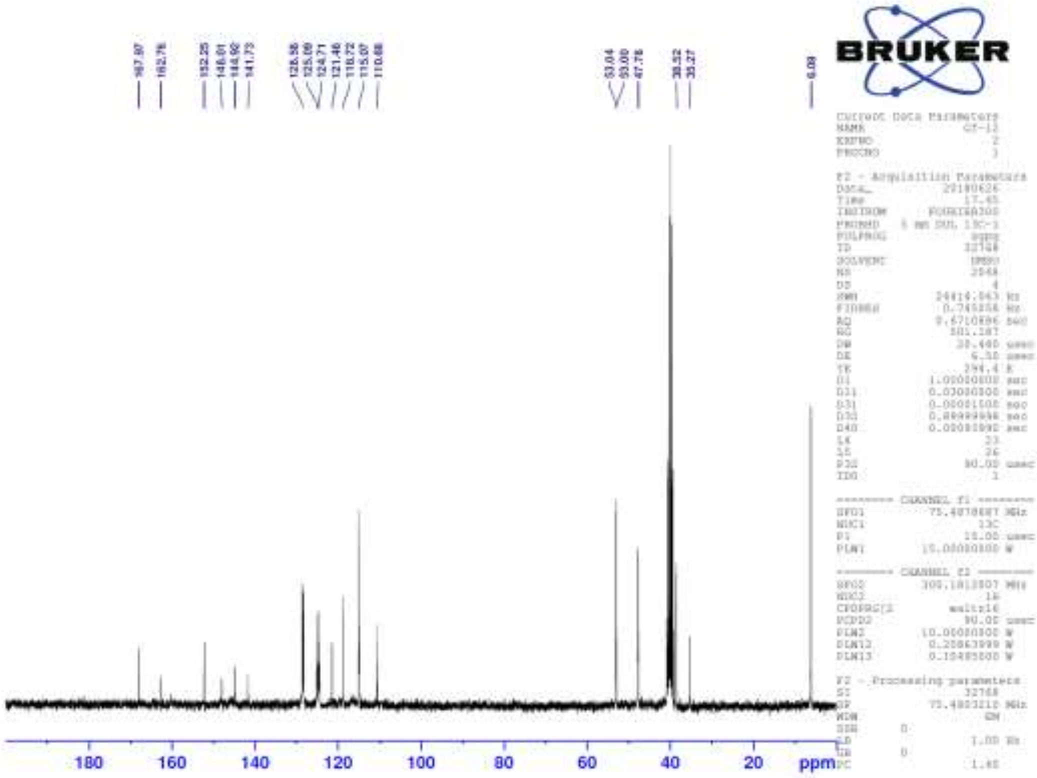

Spectra 15. ^{13}C-NMR spectra of compound **4d**

29.06.2018 16:58:53 Page 1 / 1

LabSolutions Analysis Report

<Sample Information>

Sample Name	: Bl-siklo		
Sample ID	:		
Data Filename	: Bl-siklo__001.lcd		
Method Filename	: genel.lcm		
Batch Filename	: batch.lcb		
Vial #	: 1-7	Sample Type	: Unknown
Injection Volume	: 0,1 uL		
Date Acquired	: 29.06.2018 16:34:52	Acquired by	: System Administrator
Date Processed	: 29.06.2018 16:43:33	Processed by	: System Administrator

<Chromatogram>

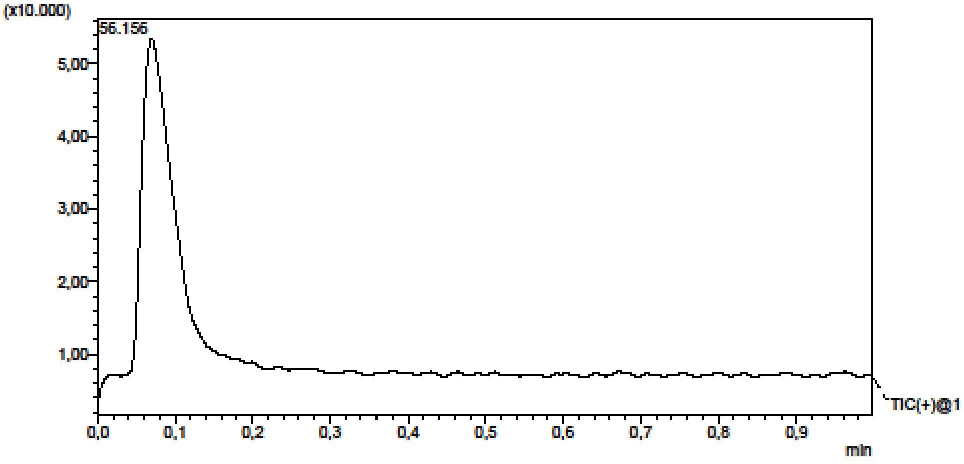

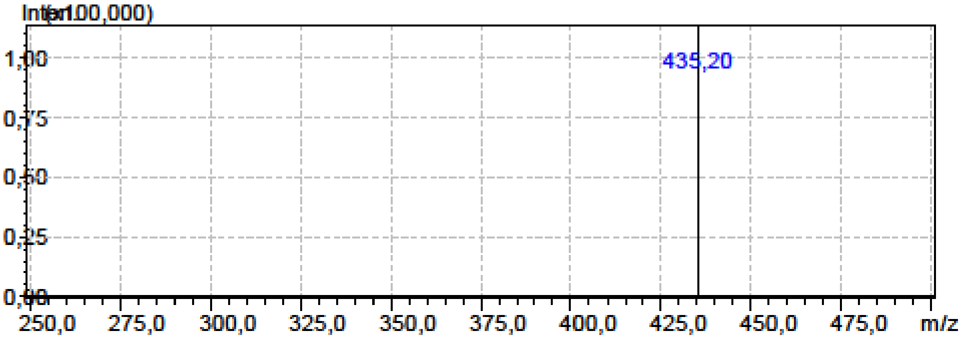

C:\LabSolutions\Data\serkan\Yeni klasör\Bl-siklo__001.lcd

Spectra 16. LCMSMS spectra of compound **4d**

DOPNALAB

	Item	Value
	Acquired Date&Time	13.04.2018 15:26:01
	Acquired by	System Administrator
	Filename	C:\Users\dopnalab\Desktop\derya\GAMZE TOKGÖZ\BT-METIL1.ispd
	Spectrum name	BT-METIL1
	Sample name	BT-METIL
	Sample ID	
	Option	
	Comment	
	No. of Scans	10
	Resolution	4 [cm-1]
	Apodization	Happ-Genzel

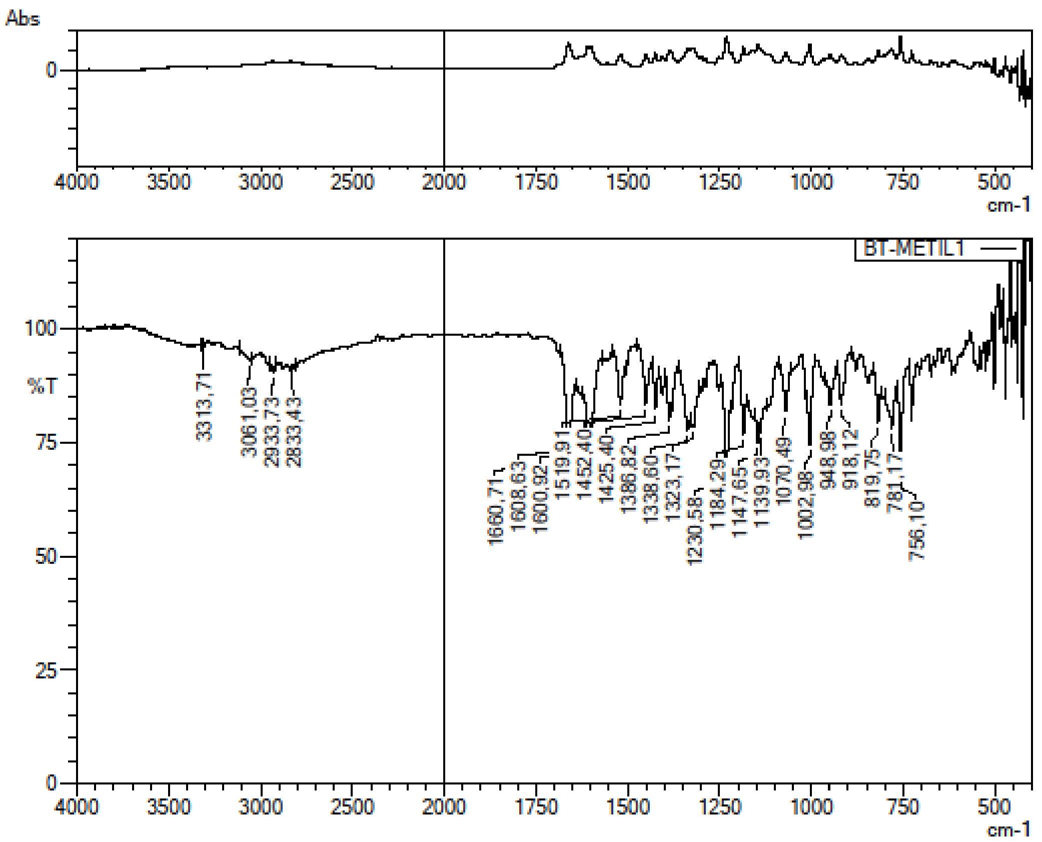

Spectra 17. IR spectra of compound **4e**

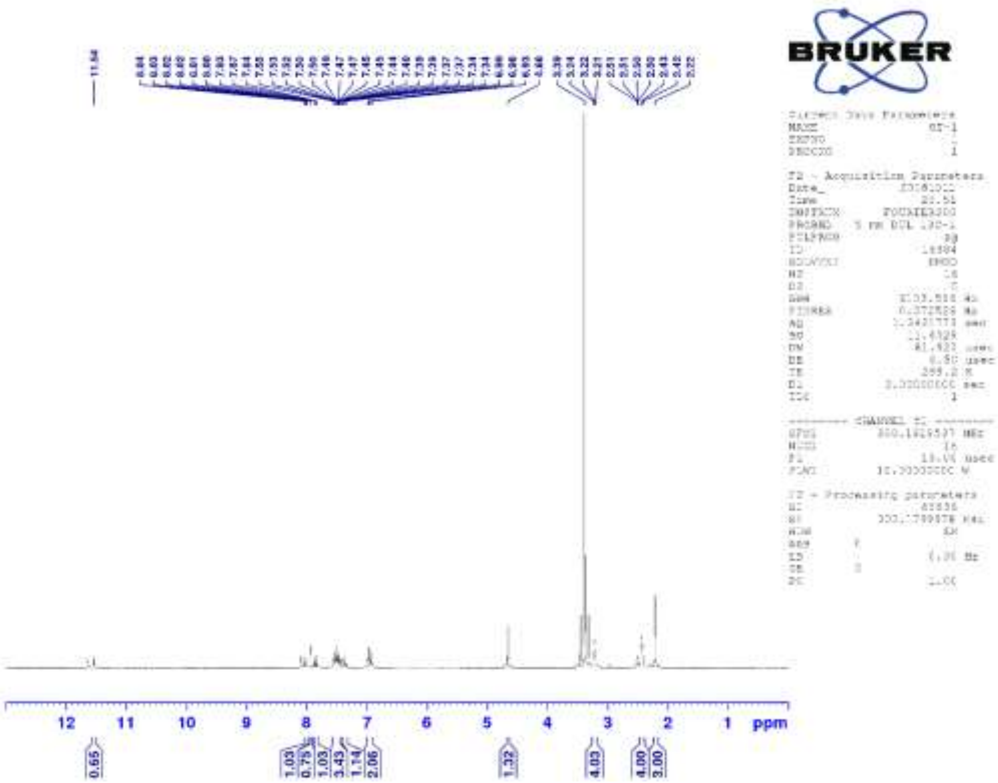

Spectra 18. ^{1}H-NMR spectra of compound **4e**

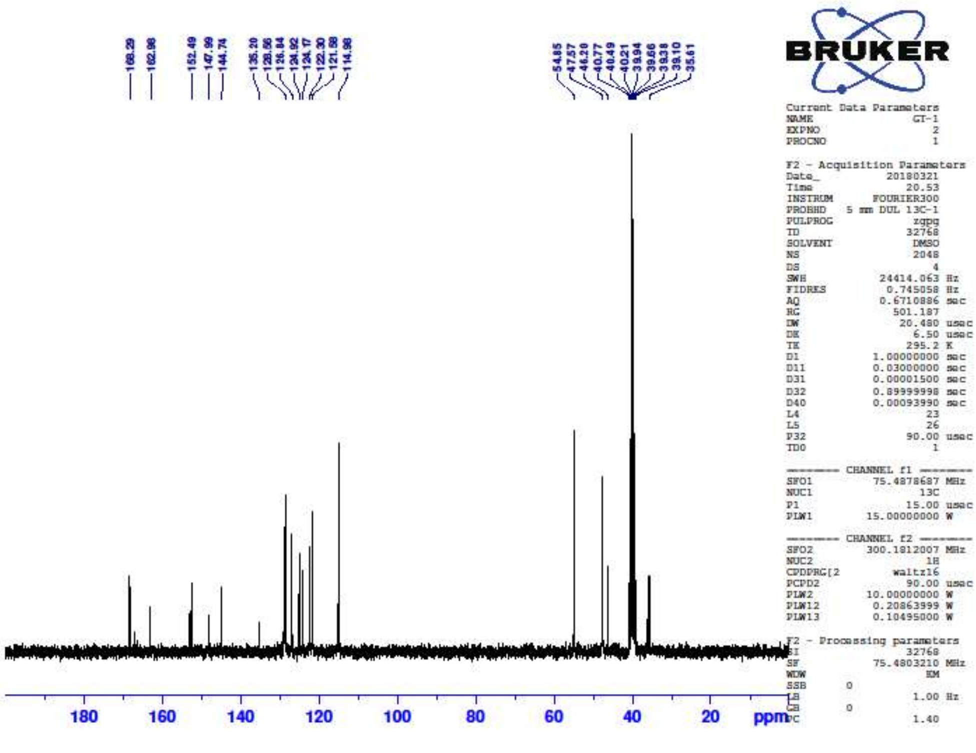

Spectra 19. ^{13}C-NMR spectra of compound **4e**

==== Shimadzu LCMSsolution Analysis Report ====

Acquired by	: Admin
Sample Name	: BT-Me
Sample ID	:
Vail #	: 12
Injection Volume	: 0.3 uL
Data File Name	: BT-Me_26.lcd
Method File Name	: genel.lcm
Batch File Name	: batch.lcb
Report File Name	: DefaultLCMS.lcr
Data Acquired	: 19.04.2018 14:13:30
Data Processed	: 19.04.2018 14:24:32

<Chromatogram>

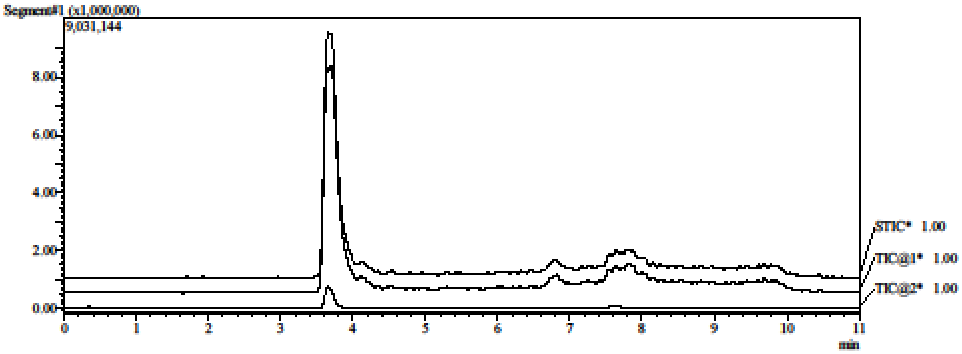

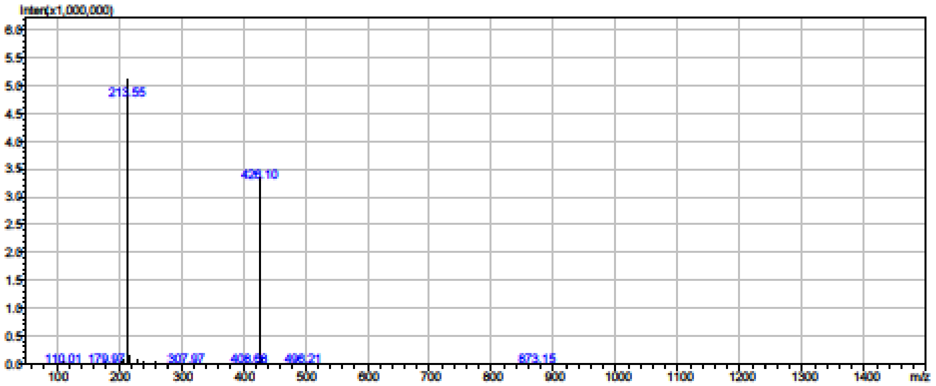

Spectra 20. LCMSMS spectra of compound **4e**

DOPNALAB

	Item	Value
	Acquired Date&Time	13.04.2018 15:28:48
	Acquired by	System Administrator
	Filename	C:\Users\dopnalab\Desktop\derya\GAMZE TOKGÖZ\BT-ET1.ispd
	Spectrum name	BT-ET1
	Sample name	BT-ET
	Sample ID	
	Option	
	Comment	
	No. of Scans	10
	Resolution	4 [cm-1]
	Apodization	Happ-Genzel

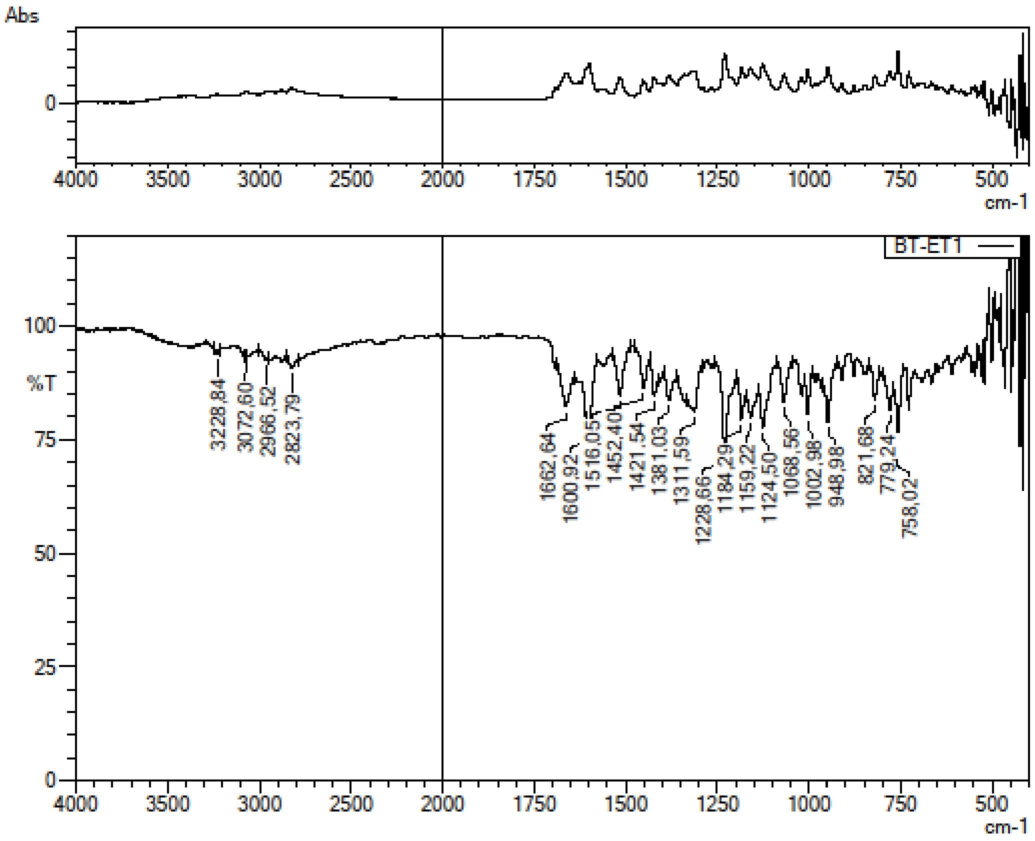

Spectra 21. IR spectra of compound **4f**

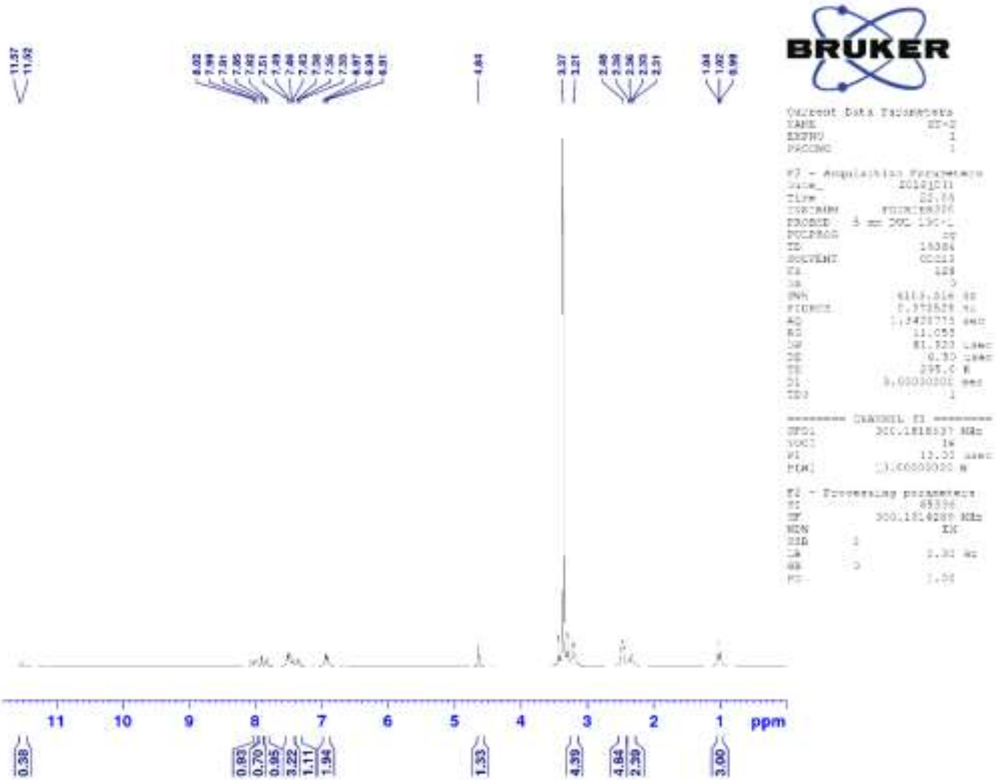

Spectra 22. ^{1}H-NMR spectra of compound **4f**

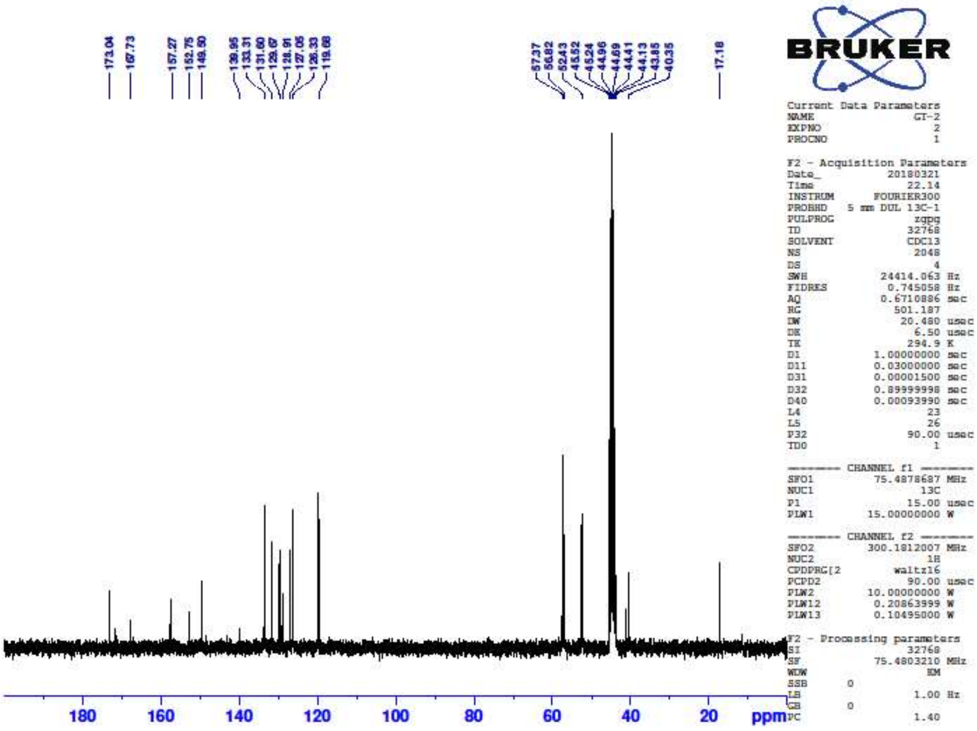

Spectra 23. ^{13}C-NMR spectra of compound **4f**

==== Shimadzu LCMSsolution Analysis Report ====

Acquired by	: Admin
Sample Name	: BT-Et
Sample ID	:
Vail #	: 10
Injection Volume	: 0.3 uL
Data File Name	: BT-Et_24.lcd
Method File Name	: genel.lcm
Batch File Name	: batch.lcb
Report File Name	: DefaultLCMS.lcr
Data Acquired	: 19.04.2018 13:50:23
Data Processed	: 19.04.2018 14:01:25

<Chromatogram>

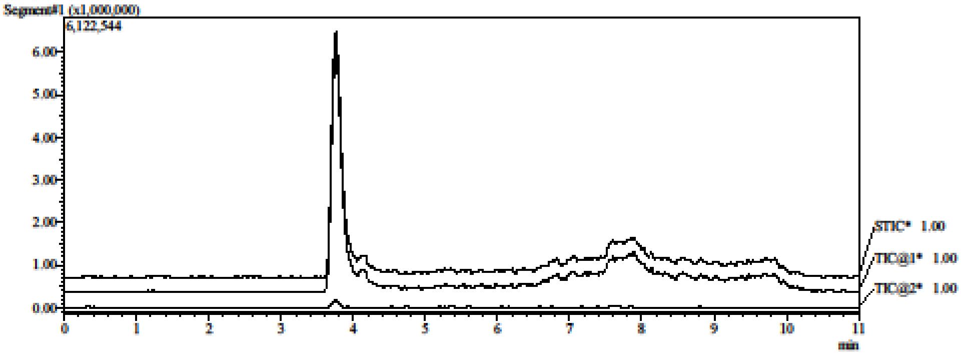

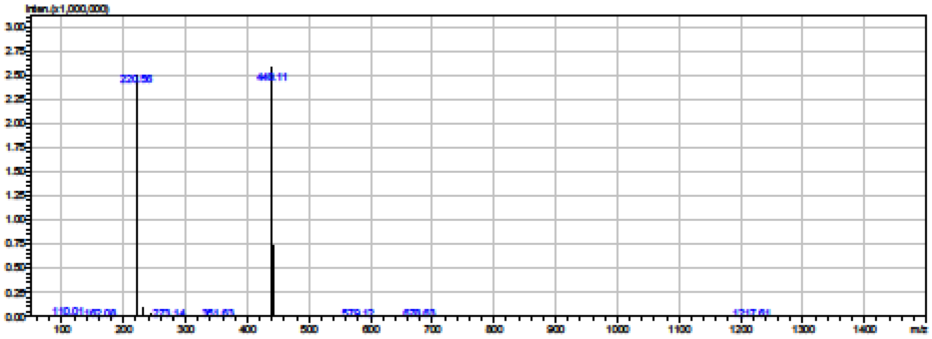

C:\LabSolutions\Data\Analiz\derya\BT-Et_24.lcd

Spectra 24. LCMSMS spectra of compound **4f**

DOPNALAB

	Item	Value
	Acquired Date&Time	13.04.2018 15:32:11
	Acquired by	System Administrator
	Filename	C:\Users\dopnalab\Desktop\derya\GAMZE TOKGÖZ\BT-ISO1.ispd
	Spectrum name	BT-ISO1
	Sample name	BT-ISO
	Sample ID	
	Option	
	Comment	
	No. of Scans	10
	Resolution	4 [cm-1]
	Apodization	Happ-Genzel

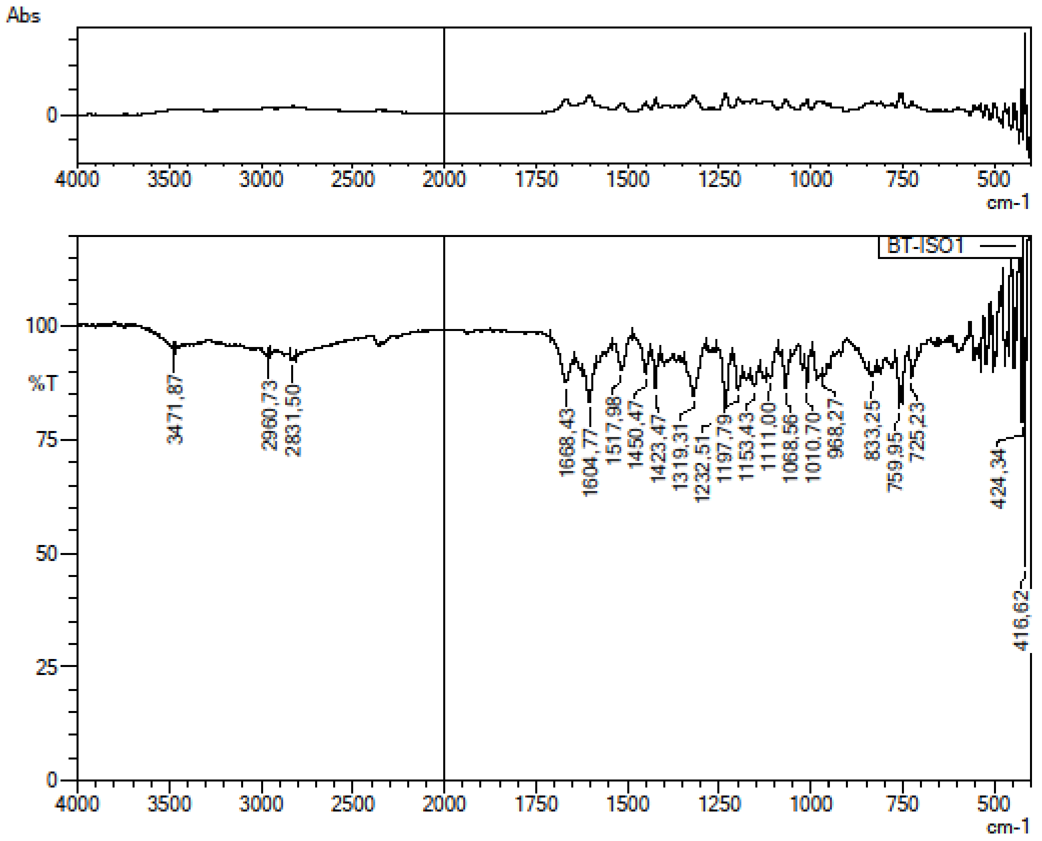

Spectra 25. IR spectra of compound **4g**

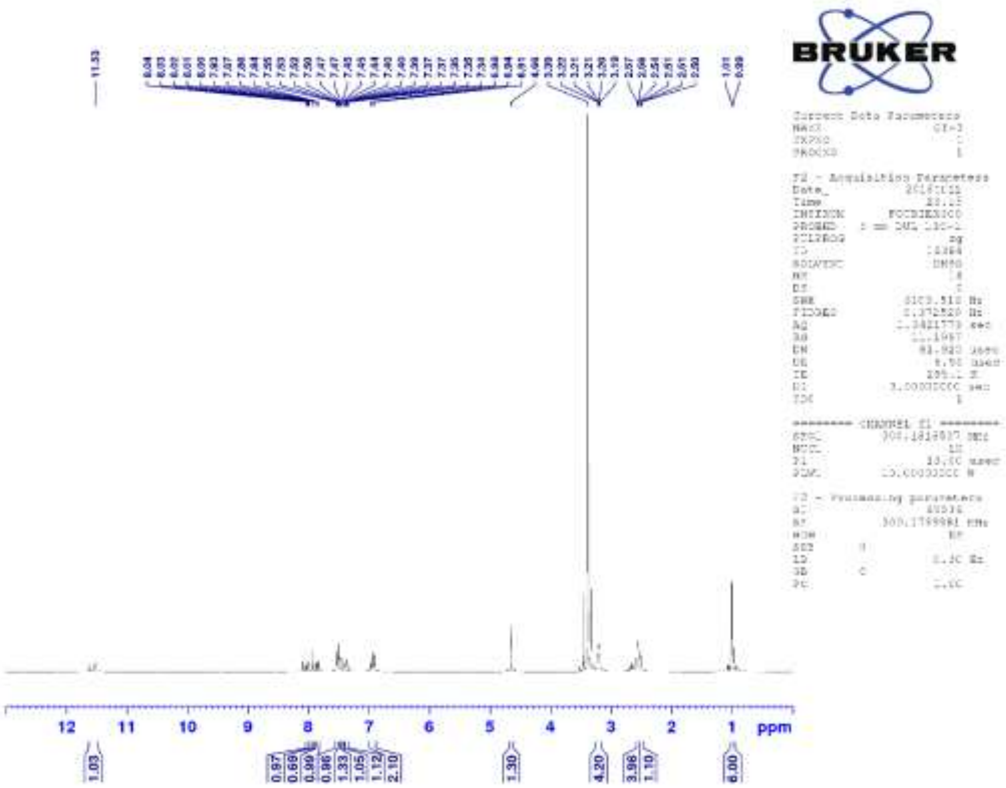

Spectra 26. ^{1}H-NMR spectra of compound **4g**

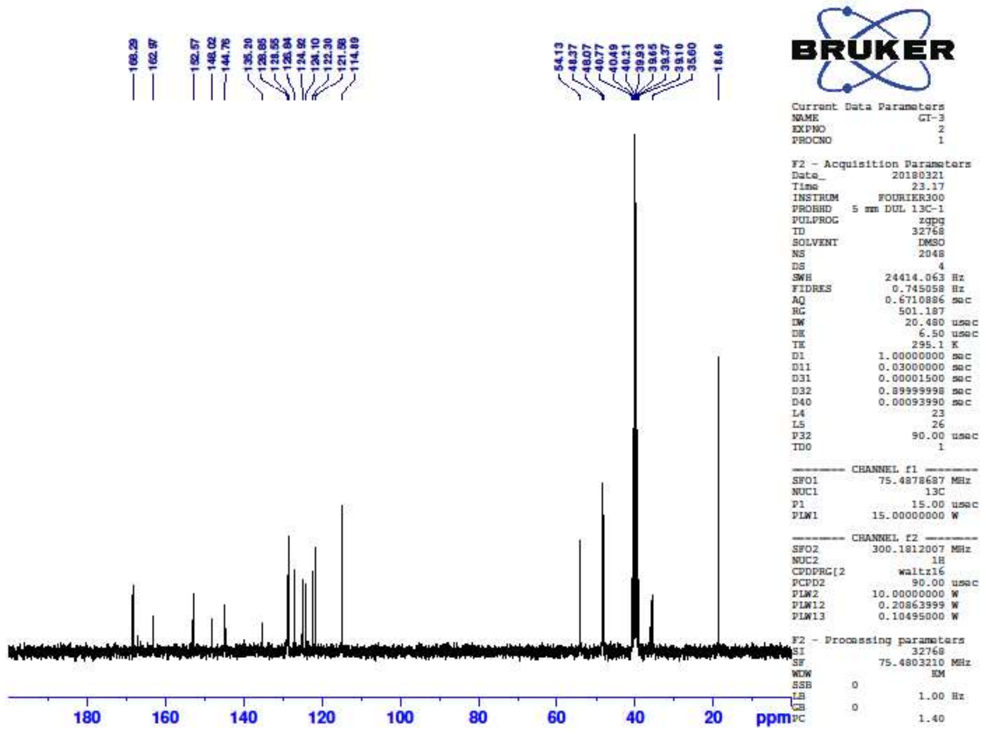

Spectra 27. ^{13}C-NMR spectra of compound **4g**

==== Shimadzu LCMSsolution Analysis Report ====

Acquired by	: Admin
Sample Name	: BT-Iso
Sample ID	:
Vail #	: 11
Injection Volume	: 0.3 uL
Data File Name	: BT-Iso_25.lcd
Method File Name	: genel.lcm
Batch File Name	: batch.lcb
Report File Name	: DefaultLCMS.lcr
Data Acquired	: 19.04.2018 14:01:56
Data Processed	: 19.04.2018 14:12:58

<Chromatogram>

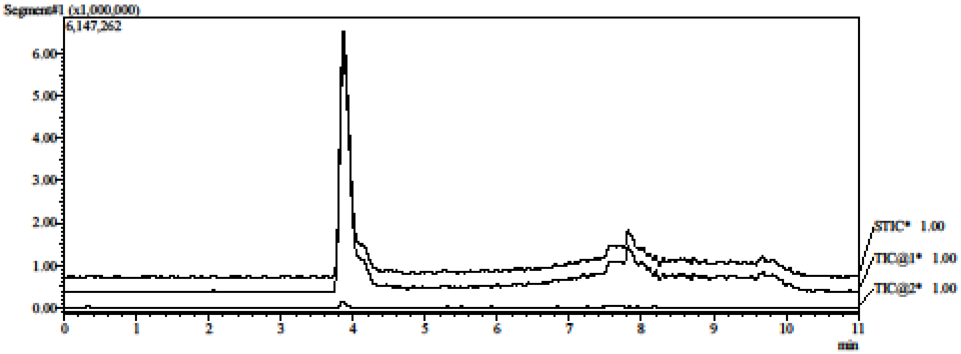

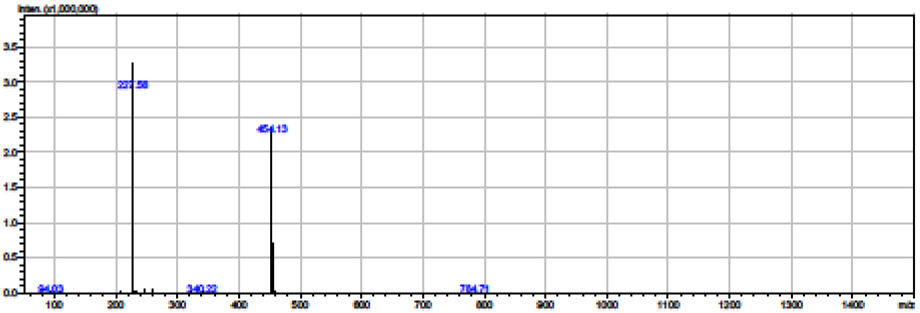

Spectra 28. LCMSMS spectra of compound **4g**

DOPNALAB

	Item	Value
	Acquired Date&Time	13.04.2018 15:34:55
	Acquired by	System Administrator
	Filename	C:\Users\dopnalab\Desktop\derya\GAMZE TOKGÖZ\BT-CYL1.ispd
	Spectrum name	BT-CYL1
	Sample name	BT-CYL
	Sample ID	
	Option	
	Comment	
	No. of Scans	10
	Resolution	4 [cm-1]
	Apodization	Happ-Genzel

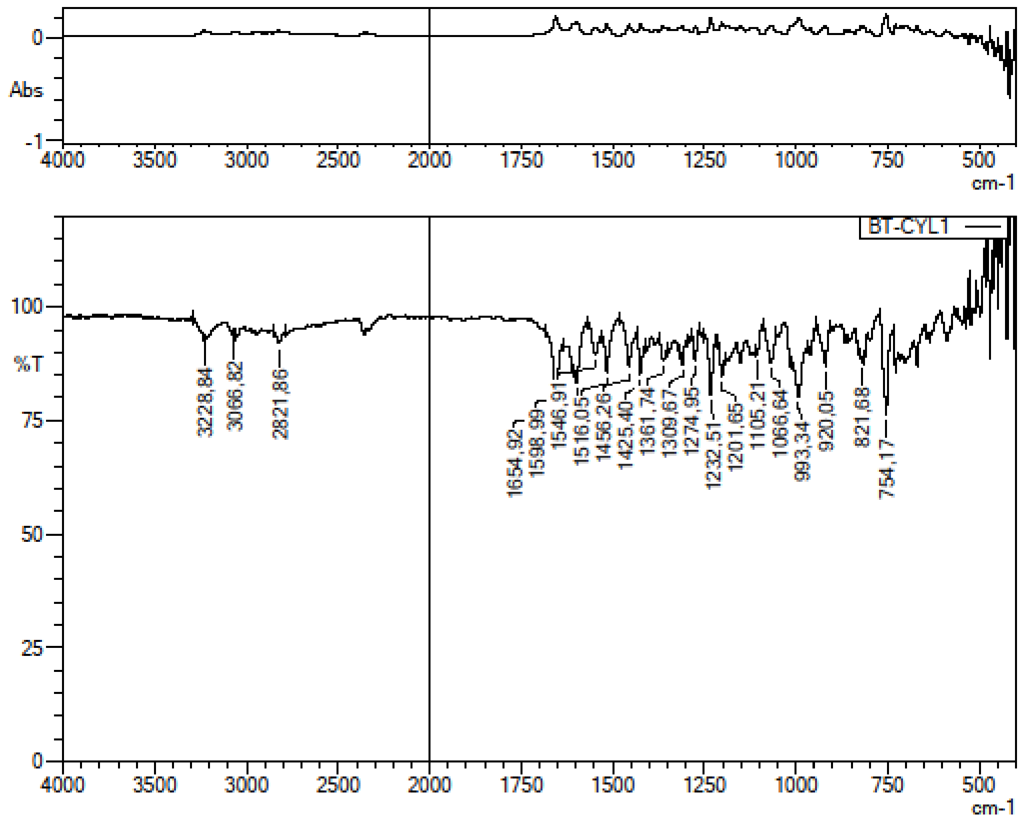

Spectra 29. IR spectra of compound **4h**

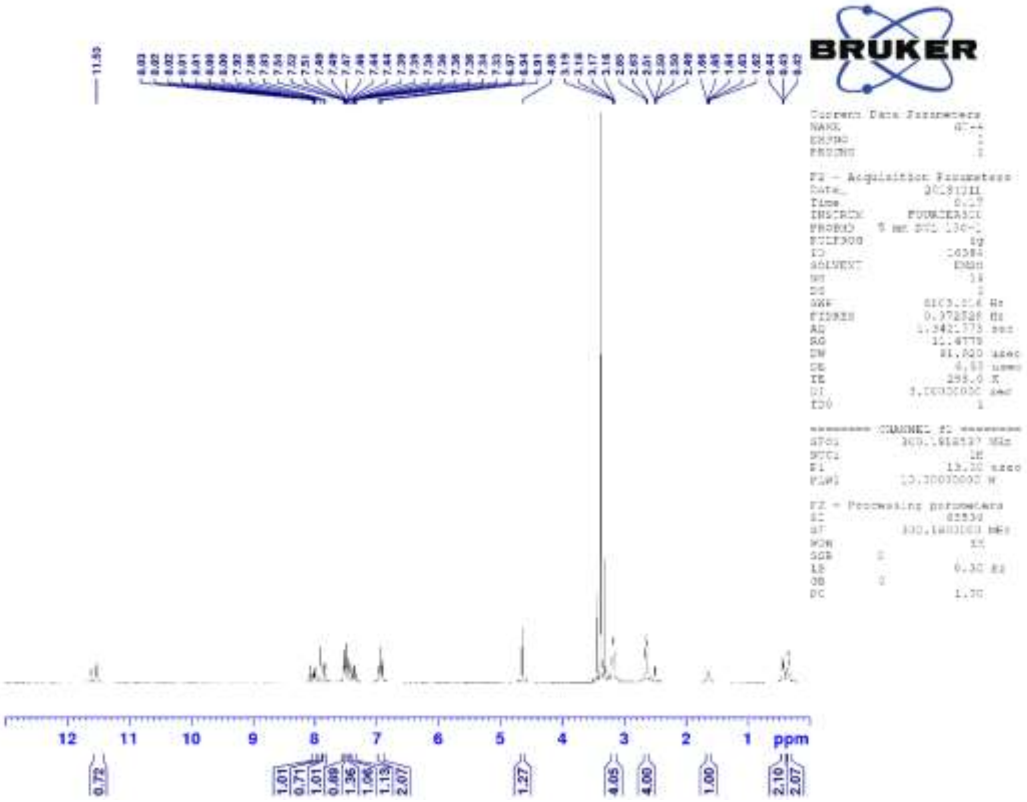

Spectra 30. ^{1}H-NMR spectra of compound **4h**

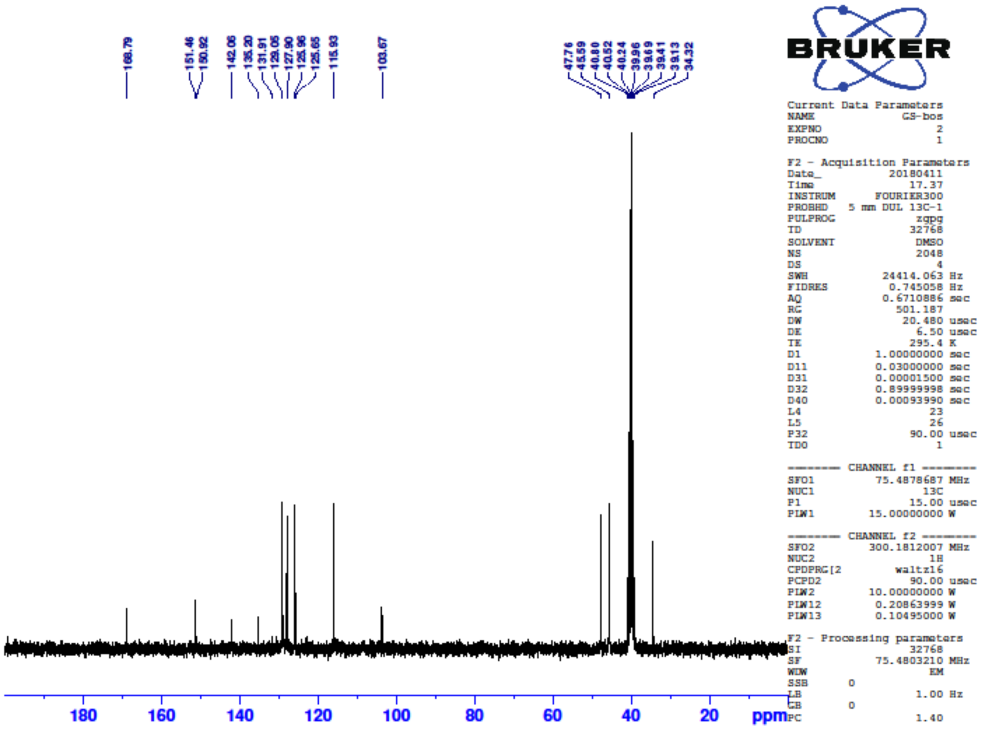

,

Spectra 31. ^{13}C-NMR spectra of compound **4h**

Shimadzu LCMSsolution Analysis Report

Acquired by	: Admin
Sample Name	: [illegible]
Sample ID	:
Vial #	: 9
Injection Volume	: [illegible] uL
Data File Name	: [illegible]
Method File Name	: [illegible]
Batch File Name	: batch.lcb
Report File Name	: DefaultLCMS.lcr
Data Acquired	: [illegible]
Data Processed	: [illegible]

<Chromatogram>

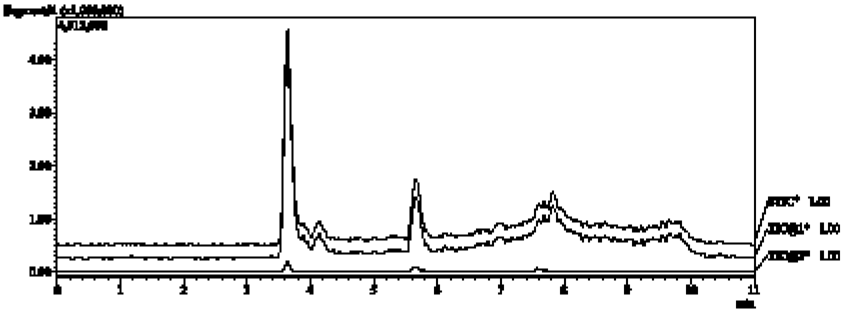

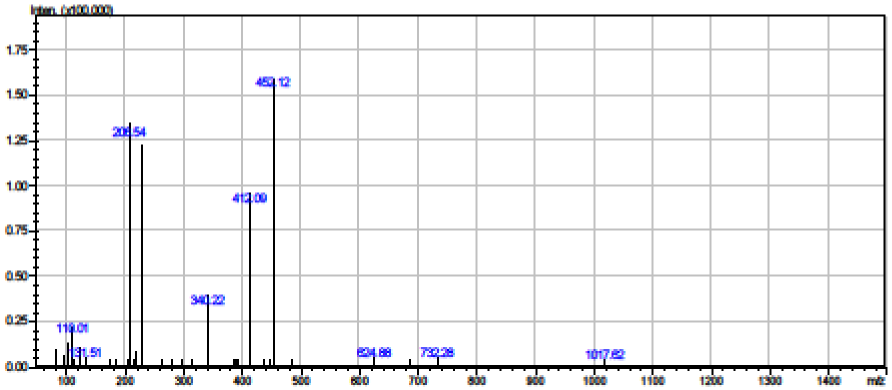

Spectra 32. LCMSMS spectra of compound **4h**
